# Cellulamides: A New Family of Marine-Sourced Linear Peptides from the Underexplored *Cellulosimicrobium* Genus

**DOI:** 10.3390/md22060268

**Published:** 2024-06-11

**Authors:** Mariana Girão, José Murillo-Alba, Jesús Martín, Ignacio Pérez-Victoria, Ricardo B. Leite, Ralph Urbatzka, Pedro N. Leão, Maria F. Carvalho, Fernando Reyes

**Affiliations:** 1 CIIMAR—Interdisciplinary Centre of Marine and Environmental Research, Terminal de Cruzeiros do Porto de Leixões, University of Porto, 4450-208 Matosinhos, Portugal; rurbatzka@ciimar.up.pt (R.U.); pleao@ciimar.up.pt (P.N.L.); mcarvalho@ciimar.up.pt (M.F.C.); 2 ICBAS—School of Medicine and Biomedical Sciences, University of Porto, 4050-313 Porto, Portugal; 3 Fundación MEDINA, Centro de Excelencia en Investigación de Medicamentos Innovadores en Andalucía, Parque Tecnológico de Ciencias de la Salud, 18016 Armilla, Spain; jose.murillo@medinaandalucia.es (J.M.-A.); jesus.martin@medinaandalucia.es (J.M.); ignacio.perez-victoria@medinaandalucia.es (I.P.-V.); 4 Genomics Unit, Instituto Gulbenkian de Ciência, 2780-156 Oeiras, Portugal; rleite@igc.gulbenkian.pt

**Keywords:** Actinomycetota, *Cellulosimicrobium*, cellulamide, linear peptides, natural products, macroalgae-associated

## Abstract

Bioprospecting the secondary metabolism of underexplored Actinomycetota taxa is a prolific route to uncover novel chemistry. In this work, we report the isolation, structure elucidation, and bioactivity screening of cellulamides A and B (**1** and **2**), two novel linear peptides obtained from the culture of the macroalga-associated *Cellulosimicrobium funkei* CT-R177. The host of this microorganism, the Chlorophyta *Codium tomentosum*, was collected in the northern Portuguese coast and, in the scope of a bioprospecting study focused on its associated actinobacterial community, strain CT-R177 was isolated, taxonomically identified, and screened for the production of antimicrobial and anticancer compounds. Dereplication of a crude extract of this strain using LC-HRMS(/MS) analysis unveiled a putative novel natural product, cellulamide A (**1**), that was isolated following mass spectrometry-guided fractionation. An additional analog, cellulamide B (**2**) was obtained during the chromatographic process and chemically characterized. The chemical structures of the novel linear peptides, including their absolute configurations, were elucidated using a combination of HRMS, 1D/2D NMR spectroscopy, and Marfey’s analysis. Cellulamide A (**1**) was subjected to a set of bioactivity screenings, but no significant biological activity was observed. The cellulamides represent the first family of natural products reported from the Actinomycetota genus *Cellulosimicrobium*, showcasing not only the potential of less-explored taxa but also of host-associated marine strains for novel chemistry discovery.

## 1. Introduction

The phylum Actinomycetota, formerly known as Actinobacteria [1], is recognized as the major microbial source of bioactive natural products (NPs), with the genus *Streptomyces* contributing by itself nearly 50% of all clinically used antibiotics [2]. However, the discovery of novel actinobacterial secondary metabolites, which have been studied for decades for their useful bioactive properties, has slowed down in part due to the frequent rediscovery of known compounds. This trend, however, is not consistent with genomic data which estimate that only 3% of the NPs potentially encoded in bacterial genomes have been experimentally characterized [3] and highlights the vast opportunities that still lie ahead for discovery. Examples of profitable strategies to reach chemical novelty rely on mining Actinomycetota from unique habitats, such as the marine environment [4], the development of innovative detection and screening techniques [5], and the bioprospecting of rare (non-*Streptomyces*) taxa [6]. 

The *Cellulosimicrobium* genus, proposed by Schumann et al. [7], belongs to the Micrococcales order and Promicromonosporaceae family, within the phylum Actinomycetota [8]. Currently, only six recognized species with validly published names are affiliated with this genus, described from very dissimilar sources such as soil [7,9], marine sediments [10], organic-waste compost [11], larvae gut [12], and human blood [13]. Research on *Cellulosimicrobium* was primarily focused on its enzymatic activities, particularly those involved in cellulose and other complex polysaccharide degradation [14,15,16], and its potential applications in industries such as biofuel production [17] and waste management [18]. Some studies have also investigated their pathogenicity to humans [19,20,21], highlighting the importance of understanding both their beneficial and potentially harmful roles in various environments. Unlike other actinobacterial taxa, such as *Streptomyces*, the production of NPs by this genus has been scarcely explored, representing, therefore, an untapped reservoir of novel chemistry with potential biotechnological applications.

Marine Actinomycetota have gathered attention due to their ability to synthesize molecules with unique chemical scaffolds, shaped by the exclusive natural pressures of their surroundings [22]. While some exist as free-living entities, others establish symbiotic relationships with several organisms, such as macroalgae; these associations prompt the synthesis of NPs with tailored bioactive properties [23]. *Cellulosimicrobium* strains have been retrieved from the marine environment, including as epiphytes of macroalgae [24], and recent studies have shown their antifungal and antioxidant properties [25,26]. However, to our knowledge, no NP to date has been described from the secondary metabolism of a *Cellulosimicrobium* strain.

In this study, we have explored the secondary metabolism of *Cellulosimicrobium funkei* CT-R177, a strain isolated from the tissues the green macroalgae *Codium tomentosum* collected in the rocky intertidal northern Portuguese coast [27]. Based on the bioactive properties displayed by the extract of this strain, and the opportunity for exploring rare Actinomycetota taxa for the production of novel chemistry, our goal was to isolate and elucidate the structure of novel molecules detected in the CT-R177 metabolome, representing the first reported NPs from the rare Actinomycetota genus *Cellulosimicrobium.*

## 2. Results and Discussion

### 2.1. Isolation and Taxonomy of Strain CT-R177

**Figure 1:**
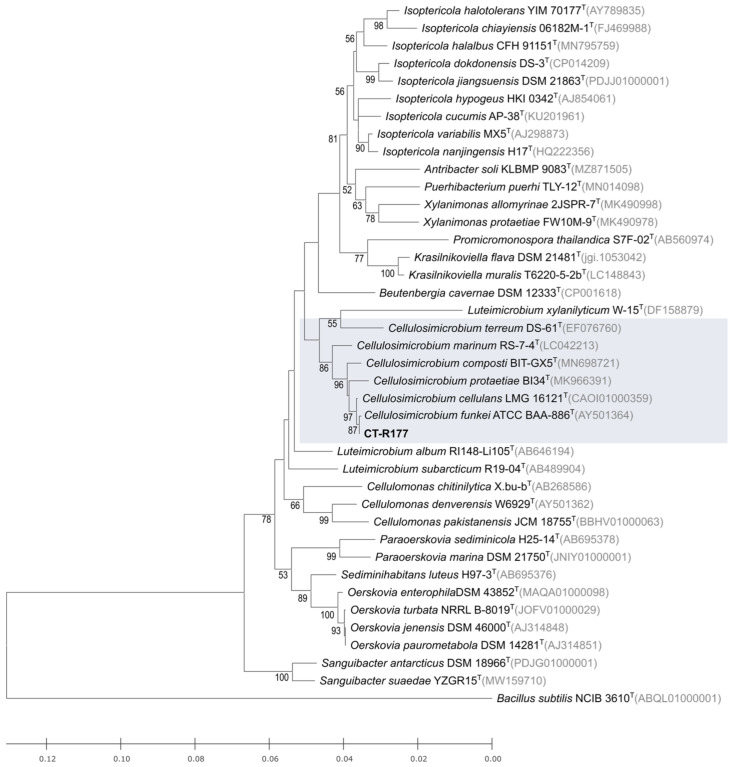
ML phylogenetic tree based on 16S rRNA gene sequences (1395 nt), showing the relationship between strain CT-R117 and the closest related type strains according to the EzBiocloud database. Accession numbers are indicated in brackets. Values at the nodes indicate bootstrap values of 50% and above, obtained based on 1000 resampling events. *Bacillus subtilis* NCIB 3610^T^ was used as an outgroup. Type strains affiliated to *Cellulosimicrobium* genus are blue shaded.

Strain CT-R177 was isolated from the holdfast tissues of a *Codium tomentosum* macroalgae specimen collected from the intertidal zone of a rocky beach in northern Portugal. A BLASTN search in the Eztaxon database of the PCR-amplified 16S rRNA nucleotide sequence showed that strain CT-R177 is closely related to *Cellulosimicrobium funkei* ATCC BAA-886^T^ (99.93% similarity) [13], followed by *Cellulosimicrobium cellulans* LMG 16121^T^ (99.86% similarity) [7]. An ML phylogenetic tree comprising the type strains closest related to strain CT-R177 was built (Figure 1), showing its close affiliation to C. *funkei*, strongly supported by the bootstrap value. Therefore, strain CT-R177 was taxonomically classified as belonging to the species *C. funkei.* The presented tree incorporates type strains from other genera beyond *Cellulosimicrobium*, enabling a more comprehensive exploration of its evolutionary relationships among other closely related Actinomycetota taxa. *C. funkei,* formerly *Oerskovia turbata*, was firstly isolated from a human blood sample, however strains affiliated with this species have since been isolated from various other settings, including the marine environment [26].

### 2.2. Bioactivity Screening and Dereplication of C. funkei CT-R177 Crude Extract

**Figure 2:**
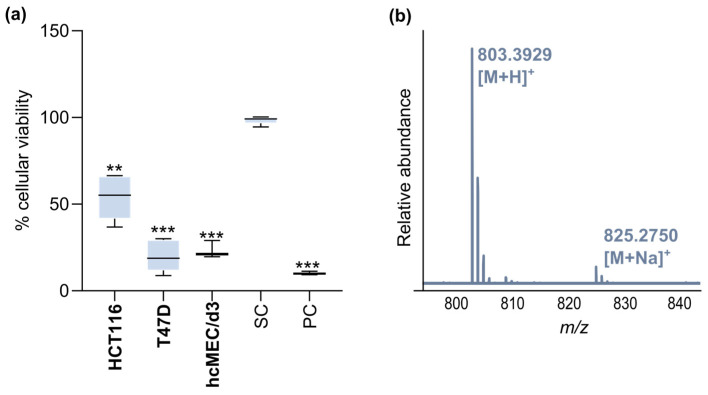
(**a**) Cytotoxic activity of strain CT-R177 crude extract in HCT116, T47D and hcMEC/d3 human cell lines, tested at 15 μg mL^−1^. Results presented as percentage of cellular viability after 48 h of exposure, measured as mean from two independent MTT experiments, each performed in triplicate. Significant differences were compared to the solvent control (** *p* < 0.01; *** *p* < 0.001). The percentage of cellular viability for the positive control (PC: Staurosporine 15 μg/mL) and solvent control (SC: 99.9% DMSO) are indicated as well. (**b**) Zoom of the HRMS spectrum displaying the *m*/*z* 803.3929 [M + H]^+^ mass spectrometric feature in strain CT-R177 crude extract.

The crude extract of strain CT-R177 was screened for antimicrobial and anticancer metabolites. While no positive result was detected against the growth of the tested microorganisms, the crude extract was able to significantly decrease the viability and proliferation of human breast ductal carcinoma T47D and colorectal carcinoma HCT116 cells (Figure 2a). However, the same effect was also detected towards the non-cancer cells, indicating general cytotoxicity rather than selectivity against cancer cell lines. In order to understand if the recorded cytotoxicity was due to any known actinobacterial compound, CT-R177 crude extract was dereplicated using GNPS tools. From this analysis, no hit with a known molecule was obtained, indicating the possible presence of a putative novel bioactive molecule in the CT-R177 metabolome. Based on this suggestion, a manual dereplication was performed to access any new mass spectrometric features of interest. One particular molecule with a protonated adduct [M + H]^+^ at *m*/*z* 803.3929 was detected, matching no accurate (exact) mass included in the Dictionary of Natural Products (DNP; dnp.chemnetbase.com, accessed on 15 March 2024), the Natural Products Atlas (NPA; npatlas.org, accessed on 15 March 2024, [28]), and Fundación MEDINA in-house database [29] (Figure 2b). Based on the molecular formula assigned of C_37_H_54_N_8_O_12_ and its likely novelty, we decided to pursue an MS-guided isolation and further chemical characterization of this molecule.

### 2.3. Mass Spectrometry-Guided Isolation and Structural Elucidation of Cellulamides

Applying a chemistry-guided isolation protocol, including reversed-phase column chromatography followed by reversed-phase HPLC, cellulamide A (**1**) and its structurally related congener cellulamide B (**2**) were isolated from the strain CT-R177 crude extract obtained with a 1:1 mixture of acetone/methanol. 

**Figure 3:**
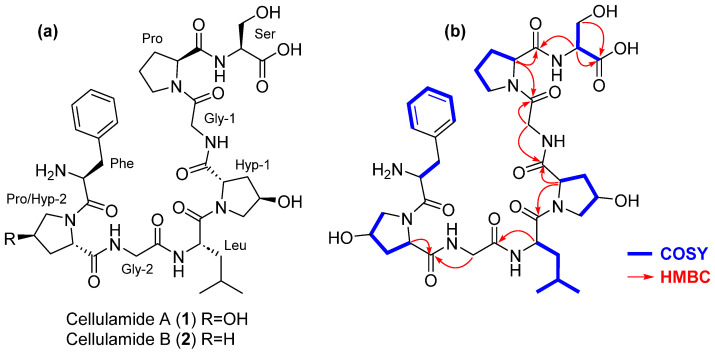
(**a**) Structure of cellulamides A and B. (**b**) Key COSY and HMBC correlations observed in the structure of cellulamide A.

**Figure 4:**
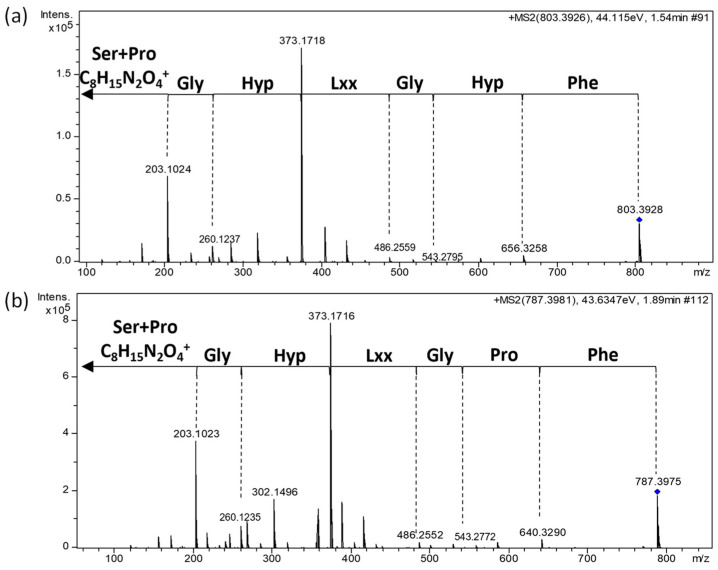
Tandem MS/MS spectra of (**a**) cellulamide A (**1**) and (**b**) cellulamide B (**2**).

A molecular formula of C_37_H_54_N_8_O_12_ was assigned to cellulamide A based on the presence of an [M + H]^+^ adduct at *m*/*z* 803.3951. Inspection of its ^1^H and ^13^C NMR spectra evidenced the peptidic nature of the compound, with a significant number of signals in the carbonyl region of the ^13^C NMR spectrum between 170 and 180 ppm combined with signals in the region corresponding to alpha hydrogens of amino acids between 3.9 and 5.0 ppm in its ^1^H NMR spectrum (Table 1, Figure S2). Additionally, signals corresponding to five hydrogens in the aromatic region of this spectrum (δ_H_ 7.24–7.32 ppm) and six carbons in the aromatic region of the ^13^C spectrum (δ_C_ 128.2 CH, 128.9 CH (×2), 131.0 CH (×2), and 138.0 C), (Table 1, Figures S2 and S3), identified Phe as one of the residues present in the molecule. Correlations observed in the COSY (Figure 3 and Figure S6) and HSQC (Figure S4) spectra additionally confirmed the presence in the structure of **1** of the following amino acids: Ser, Pro, two Gly, two γ-hydroxyproline (Hyp), and Leu. The molecular formula assigned to the compound evidenced that **1** was a linear peptide. Its sequence was established using a combination of key HMBC correlations (Figure 3 and Figure S5) together with tandem MS/MS analysis (Figure 4). The combination of both techniques established the sequence HOOC-Ser-Pro-Gly1-Hyp1-Leu-Gly2-Hyp2-Phe-NH_2_ for cellulamide A.

Marfey’s analysis was used to determine the absolute configuration of the constituent amino acids of **1** [30]. Hydrolysis of the compound followed by derivatization with N-(2,4-dinitro-5-fluorophenyl)-D-valinamide (D-FDVA) established an L configuration for the Ser, Pro, and Leu residues present in the molecule. In the case of γ-hydroxyproline, as only the *cis* and *trans* L amino acid standards were available, a double derivatization strategy using the L and D versions of FDVA was employed. This strategy unequivocally confirmed the presence of *trans* γ-L-hydroxyproline as the constituent of the molecule (see Supplementary Figures S13–S18 for Marfey’s analysis results). The full stereochemistry of the molecule was, therefore, established as depicted in Figure 3.

Having one oxygen atom less than **1**, the molecular formula of cellulamide B (**2**) was established as C_37_H_54_N_8_O_11_ based on the presence of a protonated adduct [M + H]^+^ at *m*/*z* 787.4014. Analysis of its NMR spectra evidenced that the most significant structural difference with respect to **1** was the replacement of one of the Hyp residues in **1** by a Pro in the structure of **2**. Such a change is in agreement with the difference in molecular formula observed between both compounds. Tandem MS/MS analysis (Figure 4) unequivocally established that the Hyp/Pro change was in the residue located between Gly-2 and Phe. The existence of a prominent ion at *m*/*z* 373.1718 in **1** or 373.1716 in **2**, originated from the breakage of the amide bond between Hyp-1 and Leu in both compounds, additionally supported this structural proposal. Finally, almost identical ^1^H and ^13^C NMR chemical shifts (Table 1) and a common biosynthetic origin [31] allowed us to propose the absolute configuration of cellulamide B as depicted in Figure 3. 

### 2.4. Genomic Analysis

The genome of *C. funkei* CT-R177 was sequenced and explored to better understand the biosynthetic machinery of this strain and unveil any putative BGC behind cellulamides production. The genome data (100% complete genome) were assembled into one contig with a length of 4,424,308 bp and an in silico G + C content of 74.6 mol%. Genome Taxonomy Database (GTDB) assignment [32] confirmed the taxonomic identification of strain CT-R177 as *C. funkei*, with an Average Nucleotide Identity (ANI) of 95.98% to the type strain ATCC BAA-886^T^. Differently from our prediction, antiSMASH analysis did not allow to clearly assign a BGC to the biosynthesis of the cellulamides. Also, unlike other Actinomycetota taxa that are highly enriched in BGCs, such as *Streptomyces* species that can encode up to 70 BGCs per genome [33], *C. funkei* CT-R177 appears to encode only five BGCs, three of them being assigned to peptide synthesis (Table 2). Because none of the antiSMASH-detected BGCs matched could be reasonably assigned to the biosynthesis of the cellulamides, we manually explored the genome data. Specifically, we looked for nucleotidic sequences that could translate into the amino acid sequence of **1**, and we searched also for homologs of enzymes that have been shown to be involved in proline hydroxylation a distinctive structural feature of **1**. We further looked into adenylation domains, typical of NRPSs (nonribosomal peptide synthetases—NRPSs), that could activate amino acids found in **1**. These searches did not yield any evidence that could enable us to link the genome data to the cellulamides. Thus, further studies should be conducted in the future to clarify the genetic basis of cellulamide biosynthesis.

### 2.5. Bioactivity Screening of Cellulamide A

The biological activity of cellulamide A was tested in the same antimicrobial and cytotoxic screenings previously performed with the crude extract. The compound was not active, thus proving that it was not the metabolite responsible for the previously detected anticancer activity. Cellulamide A was then tested in an additional panel of microbial human and fish pathogenic strains, including Gram-negative (*Acinetobacter baumannii*) and Gram-positive bacteria (methicillin-resistant and methicillin-sensitive *Staphylococcus aureus* and *Enterococcus faecalis*), and a yeast (*Candida albicans*). The compound was not active at the highest concentration tested of 128 μg/mL. Additionally, the compound was not cytotoxic against a panel of five human cancer cell lines when tested at a concentration of 50 μM.

## 3. Materials and Methods

### 3.1. Isolation of Strain CT-R177

Strain CT-R177 was obtained from one specimen of the Chlorophyta *Codium tomentosum*, collected in January 2020 in the intertidal area of the northern Portuguese rocky shore (41.309298°; −8.742228°), as described in Girão et al., 2024 [27]. The macroalgae was processed in the laboratory for Actinomycetota isolation and, from its holdfast tissues, strain CT-R177 was isolated in Actinomycete Isolation Agar (AIA) [27]. After 48–72 h of incubation at 28 °C, this aerobic, non-sporulating strain exhibited colonies with a small-size rod-shaped morphology and a bright yellow coloration. Stocks of the culture were preserved at −80 °C in 30% (*v*/*v*) glycerol. 

### 3.2. Taxonomic and Phylogenetic Analysis of CT-R177

Strain CT-R177 was taxonomically identified through 16S rRNA gene sequencing, as described in Girão et al., 2024 [27]. To infer the evolutionary relationship between strain CT-R177 and their closest relatives, a phylogenetic tree using the maximum-likelihood (ML) method, based on the Tamura–Nei model, was constructed using the 16S rRNA gene sequences of the described type strains closest to strain CT-R177. Multiple sequences alignment was carried out using MUSCLE [34] from within the Geneious Prime 2023.2 software package (Biomatters, Auckland, New Zealand), and 1000 bootstraps applied. MEGA-X [35] was used to build the trees. *Bacillus subtilis* NCIB 3610^T^ was used as the outgroup. 

### 3.3. Fermentation and Organic Extraction of CT-R177 Culture

A stock of strain CT-R177 was inoculated in Marine Agar (MA; Sigma-Aldrich, MO, USA) and incubated for 7 days at 28 °C. After confirming the culture purity, one colony of CT-R177 was inoculated in a 100 mL Erlenmeyer flask containing 30 mL of marine broth (MB; Sigma-Aldrich, St. Louis, MO, USA). MA and MB were used, instead of the isolation medium (AIA), since they allowed better biomass yield. The flask was incubated in an orbital shaker at 28 °C, 100 rpm, in the dark. After 4 days of incubation, 0.5 g of sterilized Amberlite XAD16N resin (Sigma-Aldrich, MO, USA) was added to the flask and incubation continued for three additional days. After this period, both biomass and resin were harvested by centrifugation (2500× *g*, 5 min), washed twice with deionized water, and freeze-dried. A 30 mL 1:1 mixture of acetone/CH₃OH was added to the lyophilizate of each culture and kept under agitation at 200 rpm for 30 min at room temperature. The organic layer was recovered by centrifugation (2500× *g*, 5 min) and dried in a rotary evaporator and the extraction procedure repeated one more time. Once the presence of a potential new compound in the crude extract resultant from this culture was confirmed, growth of strain CT-R177 was scaled up (1.5 L) and to yield 4.47 g of crude extract after evaporation of the solvent. 

### 3.4. Initial Bioactivity Screening and Dereplication of CT-R177 Crude Extract

The crude extract of CT-R177 culture was tested for antimicrobial and anticancer activity as described in Girão et al., 2019 [36]. The crude extract was tested at 1 mg/mL against a panel of Gram-positive and Gram-negative reference bacteria (*Escherichia coli* ATCC 25922, *Bacillus subtilis*, ATCC 6633, *Staphylococcus aureus* ATCC 29213, and *Salmonella enterica* ATCC 25241) and a yeast (*Candida albicans* ATCC 10231) using the disc diffusion assay. The cytotoxicity of the crude extract was assessed at 15 µg/mL in monolayer cell cultures of two human cancer lines—T47D (breast ductal carcinoma) and HCT116 (colorectal carcinoma)—using the MTT [3-(4,5-dimethylthiazol-2-yl)-2,5-diphenyltetrazolium bromide] assay. The same test was conducted using the non-cancer cell line hCMEC/D3 (human brain capillary endothelial cells). The crude extract was subjected to analysis by liquid chromatography coupled to high-resolution electrospray ionization tandem mass spectrometry (LC-HRESIMS/MS). The presence of known bioactive molecules of actinobacterial origin that could explain the recorded results was explored using the Global Natural Products Social Molecular Networking (GNPS) dereplication tools (Library Search, Dereplicator, Dereplicator VarQuest, and Dereplicator+) [37,38,39,40] following previously described methodology [36]. A potentially novel molecule with a protonated adduct [M + H]^+^ at *m*/*z* 803.3929 (**1**) was detected using this approach.

### 3.5. Mass Spectrometry-Guided Isolation and Structure Elucidation of Cellulamides

An MS-guided isolation protocol was performed to isolate the detected molecule with a protonated ion [M + H]^+^ at *m*/*z* 803.3929 (**1**). The crude extract of strain CT-R177 obtained from the 1.5 L culture was fractionated in a reverse-phase C_18_ flash chromatography using a 50 min gradient of 5–50% H_2_O/CH_3_CN, followed by 10 min at 100% CH_3_CN (CombiFlash Rf400x, Teledyne ISCO, Lincoln, NE, USA). The column was assembled in-house with 45 g (3.3 × 100 mm) of Sepra^TM^ C-18-E (50 µm, 65 Å) silica gel (Phenomenex, Torrence, CA, USA), the flow established at 10 mL/min and UV detection at 210 and 280 nm. From this separation, 40 fractions were generated and analyzed by LC-HRMS to follow the target mass. Fractions containing **1** (17 and 18, 22.6 and 15.5 mg, respectively) were individually subjected to reverse-phase HPLC using a 35 min gradient of 5–35% H_2_O/CH_3_CN. The separation was performed using an X-Bridge^TM^, XB-C18 5 µm OBD 19 × 250 mm column (Waters Corporation, Milford, MA, USA), the flow established at 10 mL/min and UV detection at 210 and 280 nm. The same chromatographic method was applied to both fractions, generating 80 new fractions from each that were all analyzed by LC-HRMS. Fractions 17_42 and 17_43 were pooled together, yielding 3.6 mg of **1**. From fractions 18_42 and 18_43, an additional amount of **1** (3.1 mg) was obtained using the same HPLC conditions. Other fractions containing **1** (17_41, 17_44, 18_41, and 18_44) were pooled together (2.1 mg) and purified by reverse-phase semi-preparative HPLC using a 35 min gradient of 10–30% H_2_O/CH_3_CN. The separation was performed using an X-Bridge^TM^ Prep Phenyl 10 × 150 mm column from Waters, using a flow of 3 mL/min and UV detection at 210 and 280 nm. An additional amount of **1** (1.1 mg) was obtained in this process. Fraction 18_46 contained compound **2** (1.6 mg). 

Cellulamide A (**1**): white amorphous solid; ${\left[\alpha \right]}_{D}^{25}$—35.3 (*c* 0.1, MeOH); UV (DAD) λ_max_ no absorption; for ^1^H and ^13^C NMR data see Table 1; (+)-ESI-qTOF MS *m*/*z* 803.3951 [M + H]^+^ (calcd for C_37_H_55_N_8_O_12_^+^, 803.3934, Δ 2.1 ppm); 825.3745 [M + Na]^+^ (calcd for C_37_H_54_N_8_O_12_Na^+^, 825.3753, Δ −1.0 ppm)

Cellulamide B (**2**): white amorphous solid; ${\left[\alpha \right]}_{D}^{25}$—75.5 (*c* 0.1, MeOH); UV (DAD) λ_max_ no absorption; for ^1^H and ^13^C NMR data see Table 1; (+)-ESI-qTOF MS *m*/*z* 787.4014 [M + H]^+^ (calcd for C_37_H_55_N_8_O_11_^+^, 787.3985, Δ 3.7 ppm); 809.3803 [M + Na]^+^ (calcd for C_37_H_54_N_8_O_11_Na^+^, 809.3804, Δ −0.1 ppm)

### 3.6. Marfey’s Analysis of Cellulamide A

A sample of cellulamide A (900 μg) was dissolved in 0.9 mL of 6 N HCl and heated at 110 °C for 16 h. The crude hydrolysate was evaporated to dryness under an N_2_ stream, and the residue was dissolved in 100 μL of water. To 50 μL of hydrolysate and to an aliquot (50 μL) of a 50 mM solution of each amino acid (D and L), 20 μL of 1 M NaHCO_3_ solution and a 1% (*w*/*v*) solution (100 μL) of D-FDVA (Marfey’s reagent, N-(2,4-dinitro-5-fluorophenyl)-D-valinamide) was added. Derivatization with L-FDVA under the same conditions was additionally performed with the *cis* and *trans* L-Hyp standards. The reaction mixture was incubated at 40 °C for 60 min. After this time, the reaction was quenched by the addition of 10 μL of 1 N HCl, and the crude mixture was diluted with 200 μL of CH_3_CN and analyzed by LC-DAD on an Agilent 1260 Infinity II instrument. Separations were carried out on an Atlantis T3 column (4.6 × 100 mm, 5 μm) maintained at 40 °C. A mixture of two solvents, A (10% CH_3_CN, 90% H_2_O) and B (90% CH_3_CN, 10% H_2_O), both containing 1.3 mM ammonium formate and 1.3 mM trifluoroacetic acid, was used as the mobile phase under a linear gradient elution mode (20−40% B in 20 min, 40−60% B in 5 min, 60–100% B in 0.2 min; isocratic 100% B for 3 min) at a flow rate of 1 mL/min.

Retention times (min) for the derivatized (D-FDVA) amino acid standards under the reported conditions were: D-Ser: 6.31, L-Ser: 7.35, D-Pro: 10.24, L-Pro: 13.54, D-Phe: 18.16, L-Phe: 24.33, D-Leu: 18.12, L-Leu: 25.41, *trans* L-Hyp: 5.15, *cis* L-Hyp: 6.76 (Figures S13–S17). Retention times (min) for the derivatized (L-FDVA) standards of L-Hyp were: *trans* L-Hyp: 4.89, *cis* L-Hyp: 5.74. (Figure S17). Retention times (min) for the observed peaks in the HPLC trace of the D-FDVA-derivatized hydrolysis product of cellulamide A were as follows: L-Ser: 7.38, L-Pro: 13.49, L-Phe: 24.32, L-Leu: 25.40, *trans* L-Hyp: 5.12 (Figure S18).

### 3.7. General Experimental Procedures

Optical rotations were measured using a Jasco P-2000 polarimeter (JASCO Corporation, Tokyo, Japan). UV spectra were obtained with an Agilent 1260 DAD (Agilent Technologies, Santa Clara, CA, USA). NMR spectra were recorded on a Bruker Avance III spectrometer (500 and 125 MHz for ^1^H and ^13^C NMR, respectively) equipped with a 1.7 mm TCI MicroCryoProbe^TM^ (Bruker Biospin, Falländen, Switzerland). Chemical shifts were reported in ppm using the signals of the residual solvent as an internal reference (δ_H_ 3.31 ppm and δ_C_ 49.15 ppm for CD_3_OD and CD_3_OH). LC-DAD and LC-HRMS(/MS) analysis were performed as described previously [41].

### 3.8. Genome Sequencing and Analysis

*C. funkei* CT-R177 was cultivated in AIA for 7 days and its genomic DNA extracted using the Dneasy PowerSoil Pro Kit (Qiagen, San Diego, CA, US). Whole genome sequencing was performed in the Genomics Unit from Instituto Gulbenkian de Ciência aiming a 150× depth coverage of an estimated genome of ~4.5 Mb. Briefly, the quantity of DNA was measured using a Qubit 4 Fluorometer using Qubit dsDNA BR assay kit and the library was prepared using an in-house protocol based in Nextera XT from Illumina. The resulting library was sequenced on a NextSeq 2000 from Illumina PE 150 + 150. Final assembly was established using SPAdes [42]. The completeness, contamination, and general genome statistics were determined using DFAST [43]. The genome sequence was annotated using the NCBI Prokaryotic Genome Annotation Pipeline and deposited at GenBank under the accession number SAMN41016946. AntiSMASH 7.0 [44] was used for the automated analysis and identification of secondary metabolite bio-synthetic gene clusters (BGCs) using relaxed detection settings and all extra features selected. A manual search for a putative BGCs that could be associated to with the biosynthesis of cellulamides was performed using tBlastn from within the Geneious Prime 2023.2 package.

### 3.9. Biological Activity

The biological activity of **1** was tested in all the previously described screenings performed for CT-R177 crude extract. This molecule was additionally screened for antimicrobial activity against *A. baumannii*, methicillin-resistant and methicillin-sensitive *S. aureus*, *E. faecalis*, and *C. albicans*, using established protocols [45,46]. Cellulamide A was tested using a two-fold dose response curve starting at 128 μg/mL. Cytotoxicity was additionally determined against the human cancer cell lines A2058 (melanoma), MIA PaCa-2 (pancreas), Hep G2 (liver), MCF-7 (breast), and A549 (lung) at 10 concentrations starting at 50 μM, using two-fold dose dilutions, following a reported procedure [47].

## 4. Conclusions

In this study, we have explored a seaweed symbiotic Actinomycetota for novel chemistry discovery, resulting in the isolation and structure elucidation of a new family of linear peptides. Cellulamides constitute the first reported secondary metabolites from the *Cellulosimicrobium* genus, highlighting the so far overlooked value of this taxon for NP discovery. It also draws attention to how symbiotic associations between seaweeds and Actinomycetota might inspire the synthesis of untapped novel molecules. Further investigations should be conducted to clarify the biosynthesis and biological activity of cellulamides, which so far remain elusive. Bacterial marine-derived peptides have proven their value in several fields [48,49], underscoring the importance of fully exploring the biotechnological potential of cellulamides. Additionally, clarifying the ecological role of these peptides, particularly their interactions with the seaweed host, the surrounding environment, and microbial communities, would provide insights into their broader biological significance and practical applications.

## Figures and Tables

**Table 1:** NMR data of cellulamides A (**1**) and B (**2**) in CD_3_OD (**1**) or CD_3_OH (**2**).

		1		2	
Amino Acid	Position	δ_H_, m, J (Hz)	δ_C_, Type	δ_H_, m, J (Hz)	δ_C_, Type
Ser	α	4.22, m	58.6, CH	4.23, m	58.5, CH
	β	3.82, m, 2H	64.1, CH_2_	3.82, m, 2H	64.2, CH_2_
	CO		176.5, C		176.5, C
Pro	α	4.46, m	62.3, CH	4.46, m	62.3, CH
	β	2.17, m; 2.09, m	30.5, CH	2.17, m; 2.09, m	30.5, CH
	γ	2.07, m; 1.99, m	25.8, CH_2_	2.07, m; 1.99, m	25.7, CH_2_
	δ	3.67, m; 3.57, m	48.0, CH_2_	3.67, m; 3.57, m	47.9, CH_2_
	CO		173.9, C		173.9, C
Gly-1	α	4.13 m; 4.05, m	42.9 CH_2_	4.09 m; 4.02, m	43.0 CH_2_
	CO		170.0, C		169.7, C
Hyp-1	α	4.58, m	60.9, CH	4.57, m	60.7, CH
	β	2.25, m; 2.08, m	38.7, CH_2_	2.23, m; 2.08, m	39.0, CH_2_
	γ	4.51, m	71.2, CH	4.51, m	71.2, CH
	δ	3.79, m, 2H	56.8, CH	3.78, m, 2H	56.7, CH
	CO		174.7, C		174.7, C
Leu	α	4.80, m	51.2, CH	4.80, m	51.1, CH
	β	1.62, m, 2H	41.5, CH_2_	1.62, m, 2H	41.6, CH_2_
	γ	1.69, m	25.8, CH_2_	1.69, m	25.7, CH_2_
	δ	0.93, m	23.8, CH_3_	0.94, m	23.7, CH_3_
	δ‘	0.94, m	22.3, CH_3_	0.95, m	22.3, CH_3_
	CO		173.4, C		173.2, C
Gly-2	α	4.13, m, 3.72 m	43.7, CH_2_	4.09, m, 3.74 m	43.7, CH_2_
	CO		171.6, C		171.5, C
Pro/ Hyp-2	α	4.56, m	61.4, CH	4.37, m	62.5, CH
	β	2.19, m; 2.03, m	39.0, CH_2_	2.18, m; 1.95, m	30.3, CH_2_
	γ	4.41, m	71.2, CH	1.97, m; 1.91, m	26.2, CH_2_
	δ	3.64, m; 3.24, dd (10.7, 2.9)	56.7, CH_2_	3.64, m; 3.55, m	48.6, CH_2_
	CO		174.7, C		173.6, C
Phe	α	3.98, m	55.4, CH	4.02, m	55.3, CH
	β	3.05, dd (13.5, 6.4), 2.83, dd, (13.5, 6.7)	41.3, CH_2_	3.10, dd (13.8, 6.4), 2.86, dd, (13.8, 6.9)	41.0, CH_2_
	γ		138.0, C		137.7, C
	δ, δ‘	7.30, m	131.0, CH	7.30, m	130.9, CH
	ε, ε‘	7.32, m	129.9, CH	7.32, m	129.8, CH
	ζ	7.24, m	128.2, CH	7.25, m	128.2, CH
	CO		174.8, C		173.9, C

**Table 2:** Secondary metabolite BGCs identified by antiSMASH in the genome of *C. funkei* CT-R177.

Region	Type of Compound	Most Similar Known Cluster	Similarity (%)	MiBiG Accession	Length (nt)
2.1	NI-siderophore/T3PKS	Alkylresorcinol	100	BGC0000282	1–23,448
5.1	Terpene	7-deoxypactamycin	5	BGC0000119	216,873–237,787
7.1	Thioamide-NRP	Enteromycin	12	BGC000249	130,738–176,956
12.1	RiPP-like	NH	NH	NH	123,554–176,956
16.1	RiPP-like	NH	NH	NH	36,580–46,876

T3PKS: Type III polyketide synthase; NRP: non-ribosomal peptide; RiPP: ribosomally synthesized and post-translationally modified peptide; NH—No Hits.

## Data Availability

Data are contained within the article and Supplementary Materials.

## Supplementary Materials

The following supporting information can be downloaded at: https://www.mdpi.com/article/10.3390/md22060268/s1, Figure S1: UV and (+)-ESI-TOF spectra of cellulamide A (**1**); Figure S2: ^1^H-NMR (500 MHz, CD_3_OD) spectrum of cellulamide A (**1**) Figure S3: ^13^C-NMR (125 MHz, CD_3_OD) spectrum of cellulamide A (**1**); Figure S4: HSQC (CD_3_OD) spectrum of cellulamide A (**1**); Figure S5: HMBC (CD_3_OD) spectrum of cellulamide A (**1**); Figure S6: COSY (CD_3_OD) spectrum of cellulamide A (**1**); Figure S7: UV and (+)-ESI-TOF spectra of cellulamide B (**2**); Figure S8: ^1^H-NMR (500 MHz, CD_3_OH) spectrum of cellulamide B (**2**); Figure S9: ^13^C-NMR (125 MHz, CD_3_OH) spectrum of cellulamide B (**2**); Figure S10: HSQC (CD_3_OH) spectrum of cellulamide B (**2**); Figure S11: HMBC (CD_3_OH) spectrum of cellulamide B (**2**); Figure S12: COSY (CD_3_OH) spectrum of cellulamide B (**2**); Figure S13: HPLC traces at 210 nm of the D-FDVA adducts of Ser; Figure S14: HPLC traces at 210 nm of the D-FDVA derivatives of Pro; Figure S15: HPLC traces at 210 nm of the D-FDVA derivatives of Phe; Figure S16: HPLC traces at 210 nm of the D-FDVA derivatives of Leu; Figure S17: HPLC traces at 210 nm of the D and L-FDVA derivatives of *cis* and *trans* L-Hyp; Figure S18: HPLC trace at 210 nm of the D-FDVA derivatives of the hydrolyzate of cellulamide A (**1**). Figure S19. HPLC trace at 210 nm of the crude extract of strain CT-R177.
