# Supplementary material for: Cellulamides: A New Family of Marine-Sourced Linear Peptides from the Underexplored Cellulosimicrobium Genus
Source: Mar Drugs. 2024 Jun 11;22(6):268. doi: 10.3390/md22060268 (PMC11204466; doi:10.3390/md22060268)
Supplement: Supplementary file 1 [file marinedrugs-22-00268-s001.zip › marinedrugs-3025384-supplementary.pdf]

Supplementary information for

Cellulamides: a new Family of Marine-sourced Linear Peptides from the Underexplored *Cellulosimicrobium* Actinomycetota Genus

Mariana Girão[1,2]*, José Murillo-Alba[3], Jesús Martín[3], Ignacio Pérez-Victoria[3], Ralph Urbatzka[1], Pedro N. Leão[1], Maria F. Carvalho[1], Fernando Reyes[3]*

1 CIIMAR/CIMAR, Interdisciplinary Centre of Marine and Environmental Research, Terminal de Cruzeiros do Porto de Leixões, University of Porto, 4450-208 Matosinhos, Portugal;

2 ICBAS - School of Medicine and Biomedical Sciences, University of Porto, 4050-313 Porto, Portugal;

3 Fundación MEDINA, Centro de Excelencia en Investigación de Medicamentos Innovadores en Andalucía, Parque Tecnológico de Ciencias de la Salud, 18016 Armilla, Granada, Spain.

* Correspondence: mariana.martins@ciimar.up.pt (M.G); fernando.reyes@medinaandalucia.es (F.R.);

Supporting Information Table of Contents

Contents | Page

Figure S1. UV and (+)-ESI-TOF spectra of cellulamide A (**1**)……………………………………….…………3
Figure S2. ^{1}H-NMR (500 MHz, CD_3OD) spectrum of cellulamide A (**1**)……………………….……….….4
Figure S3. ^{13}C-NMR (125 MHz, CD_3OD) spectrum of cellulamide A (**1**)……….…………..….………….5
Figure S4. HSQC (CD_3OD) spectrum of cellulamide A (**1**)……….……………………………..….….…….6
Figure S5. HMBC (CD_3OD) spectrum of cellulamide A (**1**)……….…………………………….……..…….7
Figure S6. COSY (CD_3OD) spectrum of cellulamide A (**1**)……….………………………...………….8
Figure S7. UV and (+)-ESI-TOF spectra of cellulamide B (**2**)……………………………………………….9
Figure S8. ^{1}H-NMR (500 MHz, CD_3OH) spectrum of cellulamide B (**2**)……….………………………....10
Figure S9. ^{13}C-NMR (125 MHz, CD_3OH) spectrum of cellulamide B (**2**)…….………….…………….11
Figure S10. HSQC (CD_3OH) spectrum of cellulamide B (**2**)……….……………………………...….12
Figure S11. HMBC (CD_3OH) spectrum of cellulamide B (**2**)……….………………………………...13
Figure S12. COSY (CD_3OH) spectrum of cellulamide B (**2**)……….……………………………….……14
Figure S13. HPLC traces at 210 nm of the D-FDVA derivatives of Ser…………………………………..…15
Figure S14. HPLC traces at 210 nm of the D-FDVA derivatives of Pro……………………….…………..…16
Figure S15. HPLC traces at 210 nm of the D-FDVA derivatives of Phe……………………………………17
Figure S16. HPLC traces at 210 nm of the D-FDVA derivatives of Leu……………………………………18
Figure S17. HPLC traces at 210 nm of the D and L-FDVA derivatives of *cis* and *trans* L-Hyp……..…..19
Figure S18. HPLC trace at 210 nm of the D-FDVA derivatives of the hydrolyzate of cellulamide A (**1**).20
Figure S19. HPLC trace at 210 nm of the crude extract of strain CT-R177………………………………..21

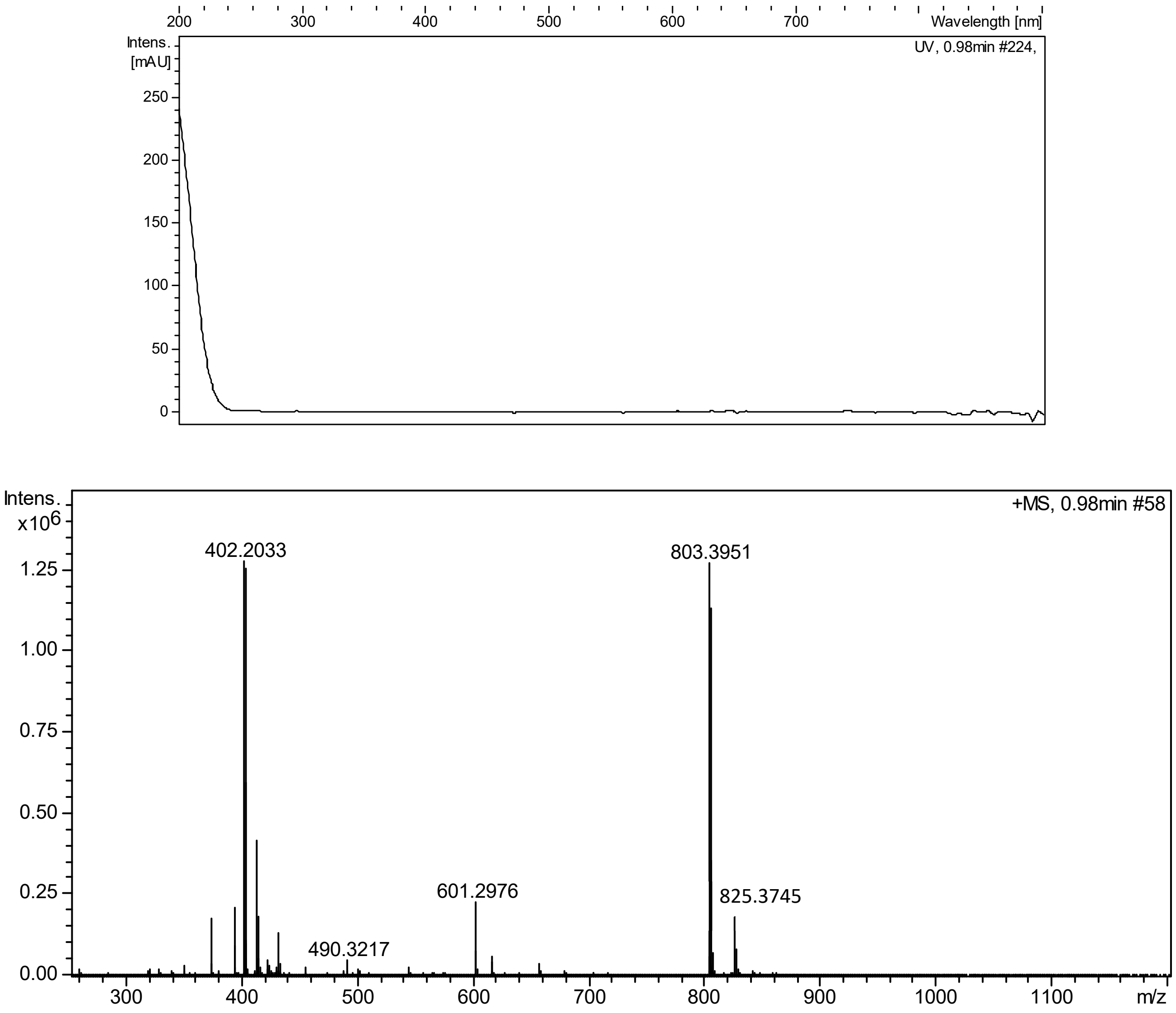

Figure S1. UV and (+)-ESI-TOF spectra of cellulamide A (**1**)

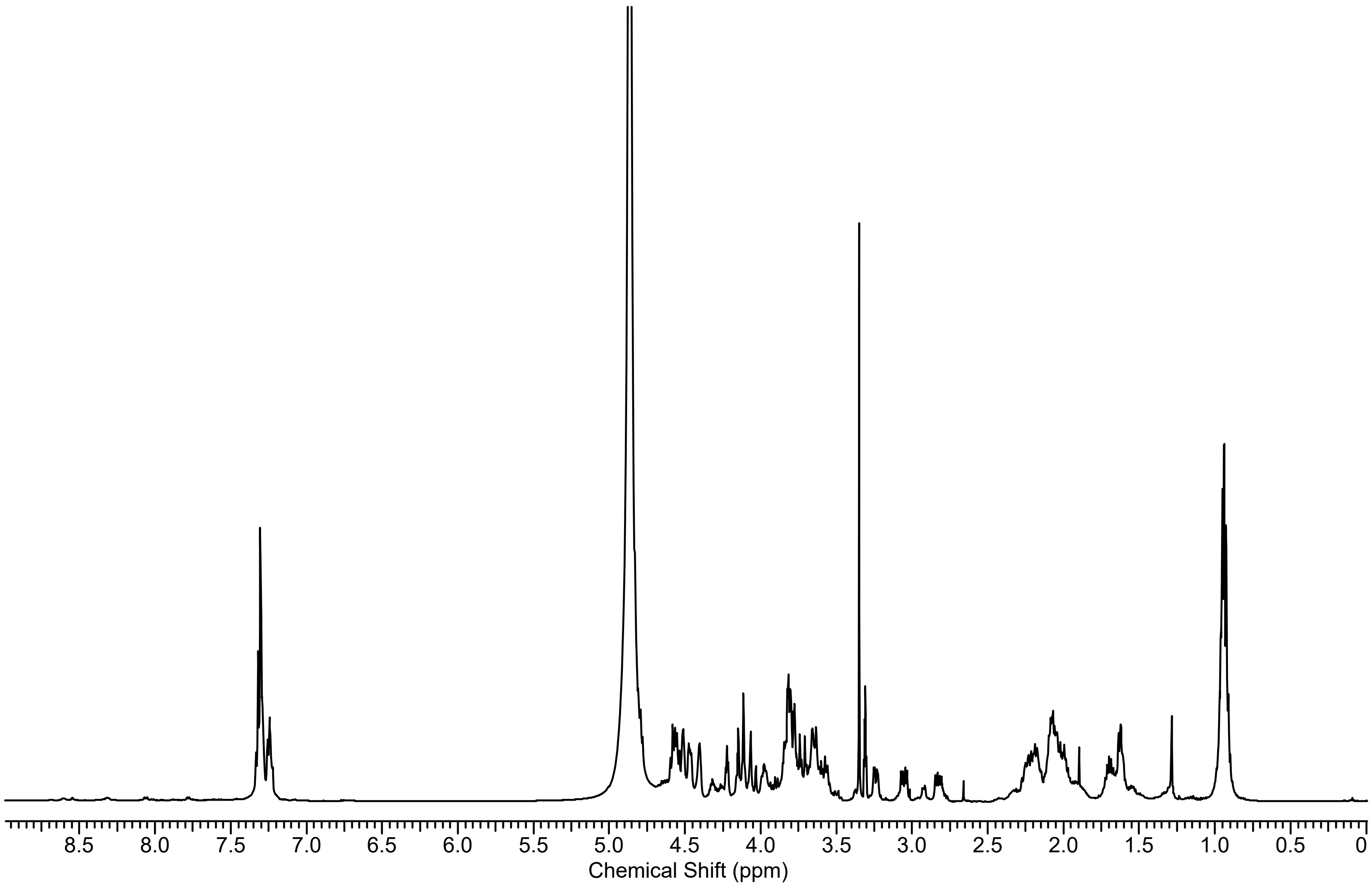

Figure S2. ^{1}H-NMR (500 MHz, CD_3OD) spectrum of cellulamide A (**1**)

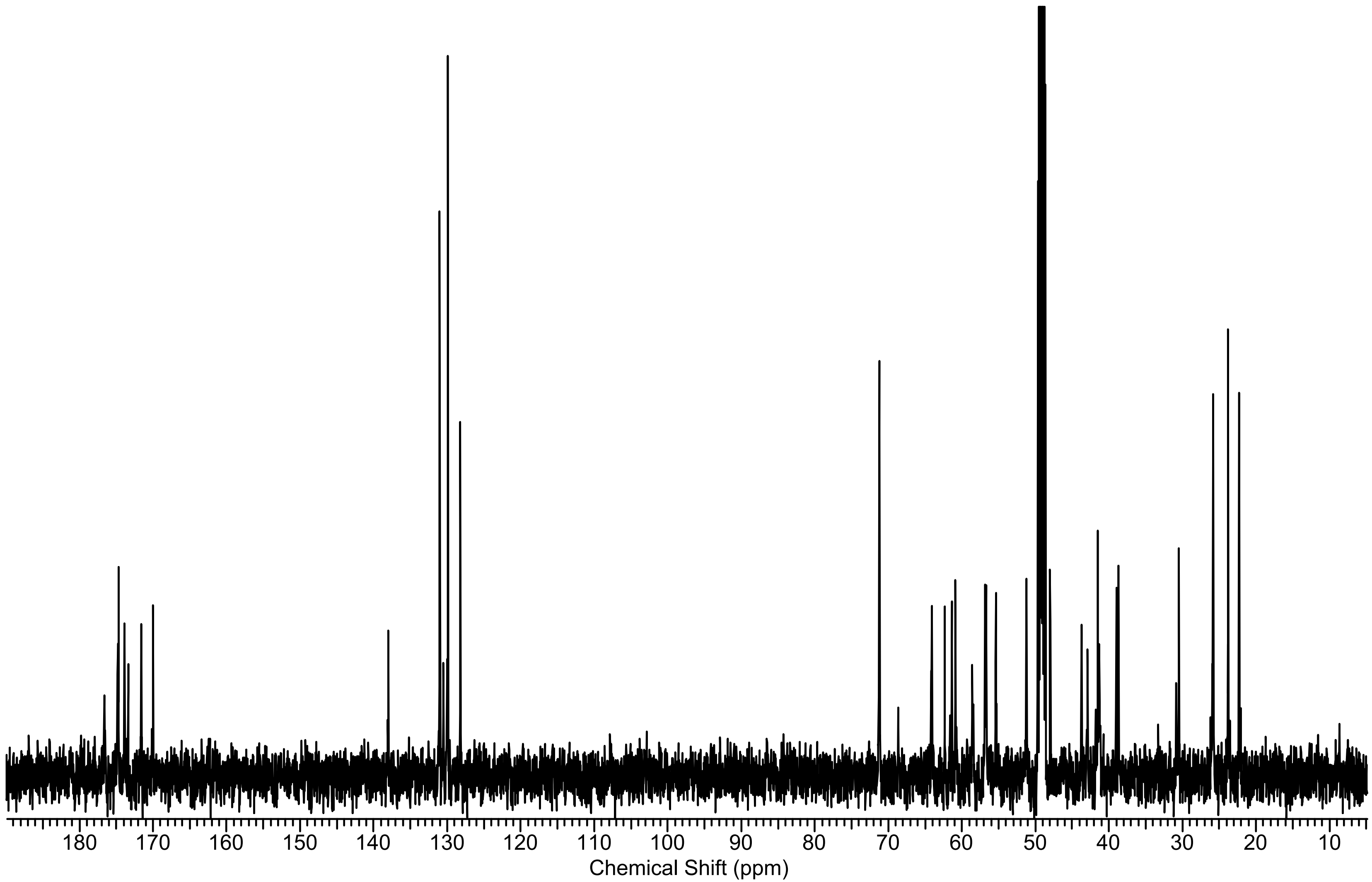

Figure S3. ^{13}C-NMR (125 MHz, CD_3OD) spectrum of cellulamide A (**1**)

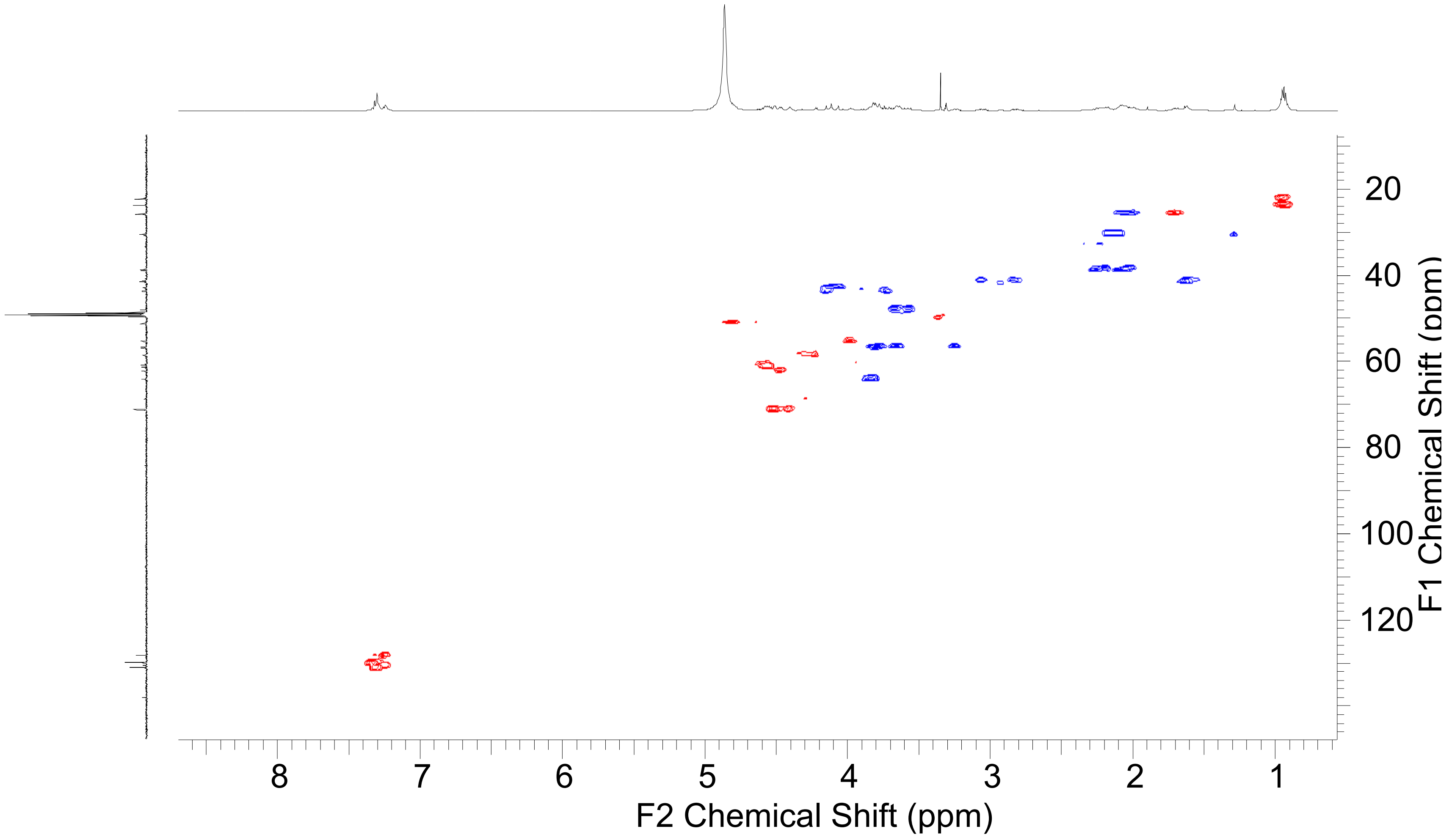

Figure S4. HSQC (CD3OD) spectrum of cellulamide A (1)

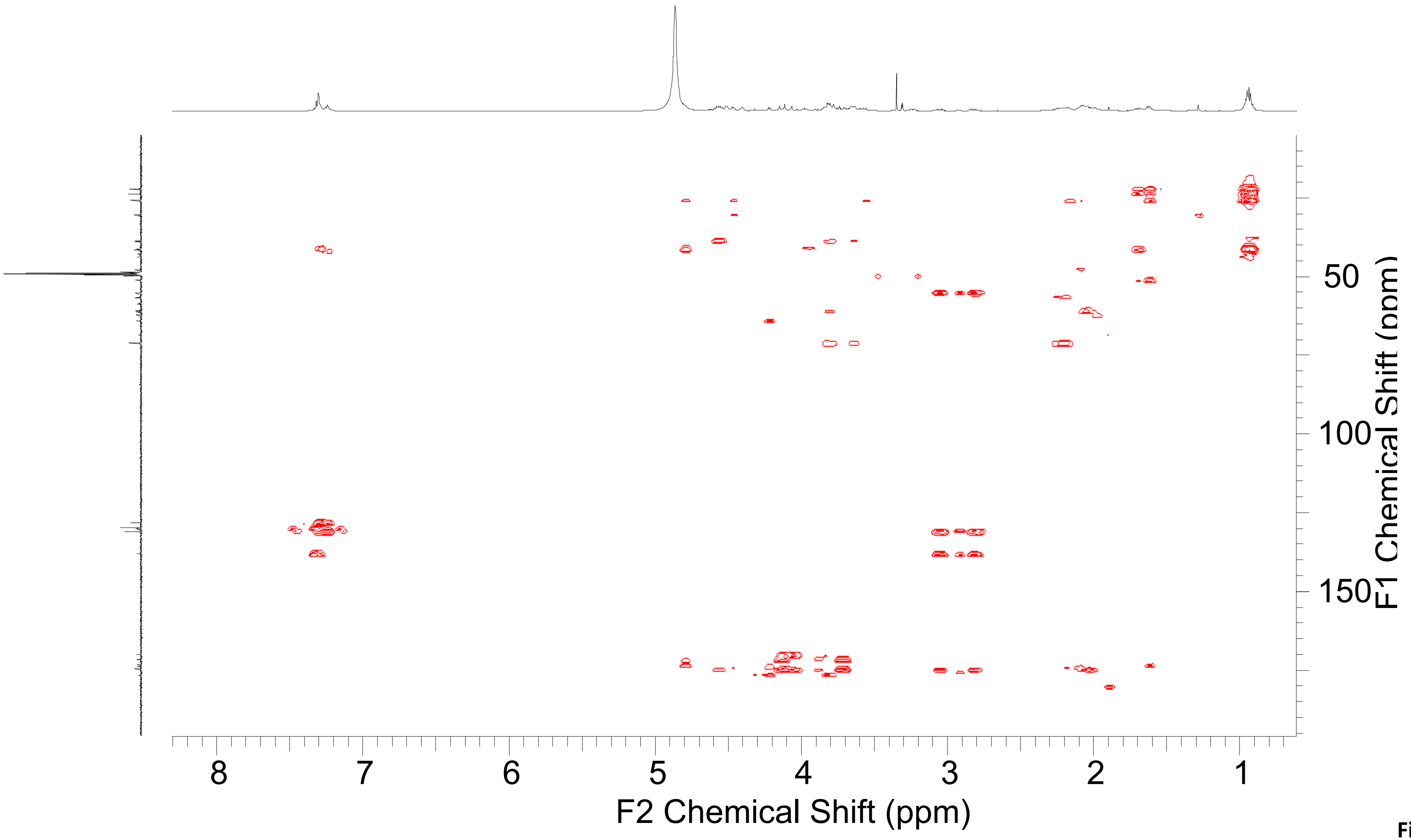

Figure

Figure S5. HMBC (CD3OD) spectrum of cellulamide A (**1**)

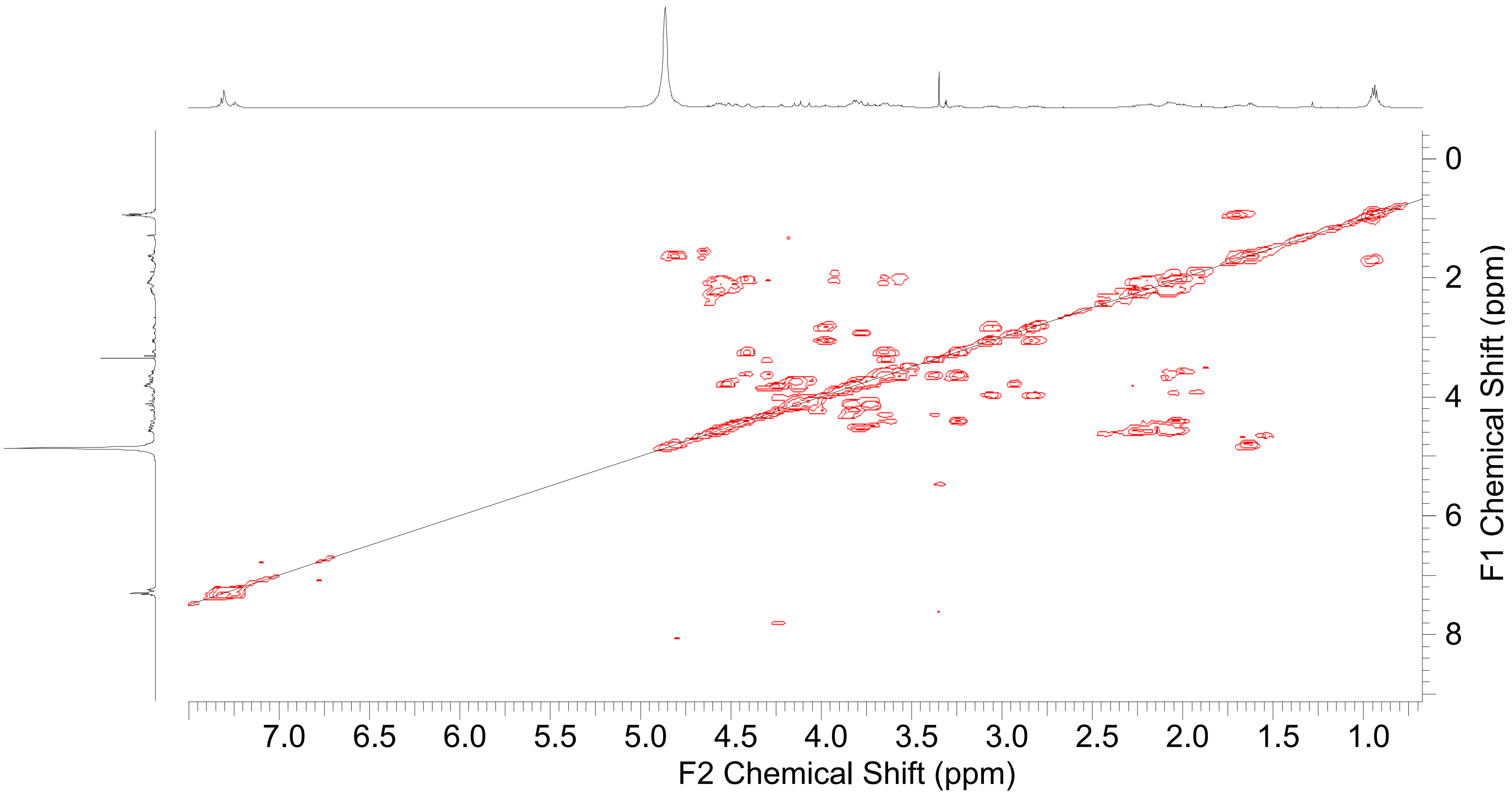

Figure S6. COSY (CD_3OD) spectrum of cellulamide A (**1**)

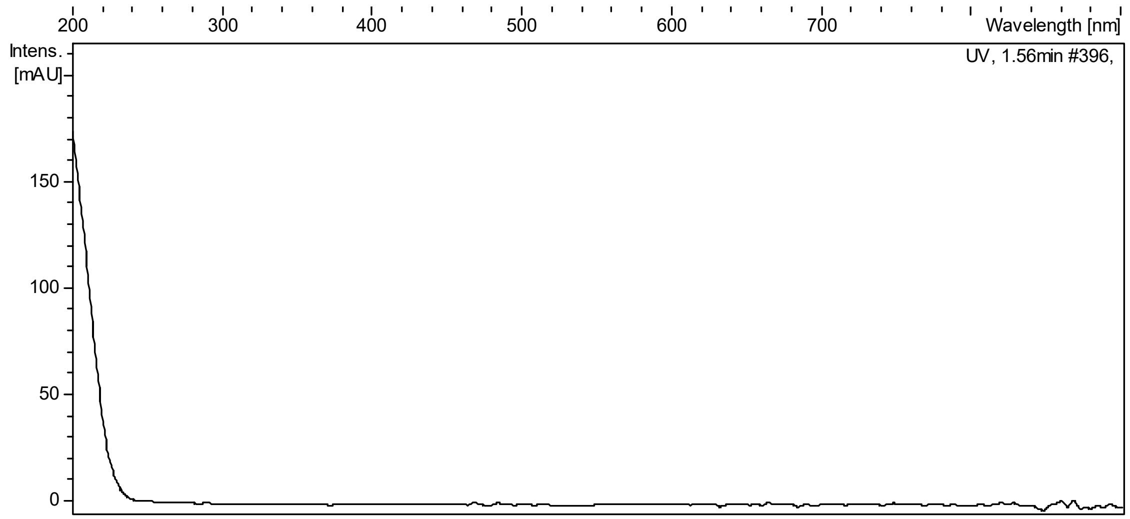

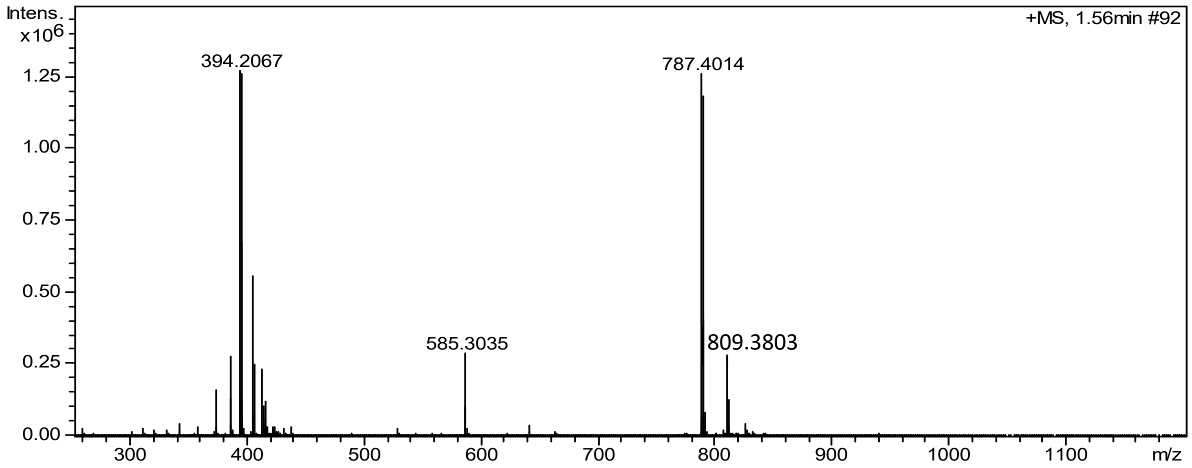

Figure S7. UV and (+)-ESI-TOF spectra of cellulamide B (**2**)

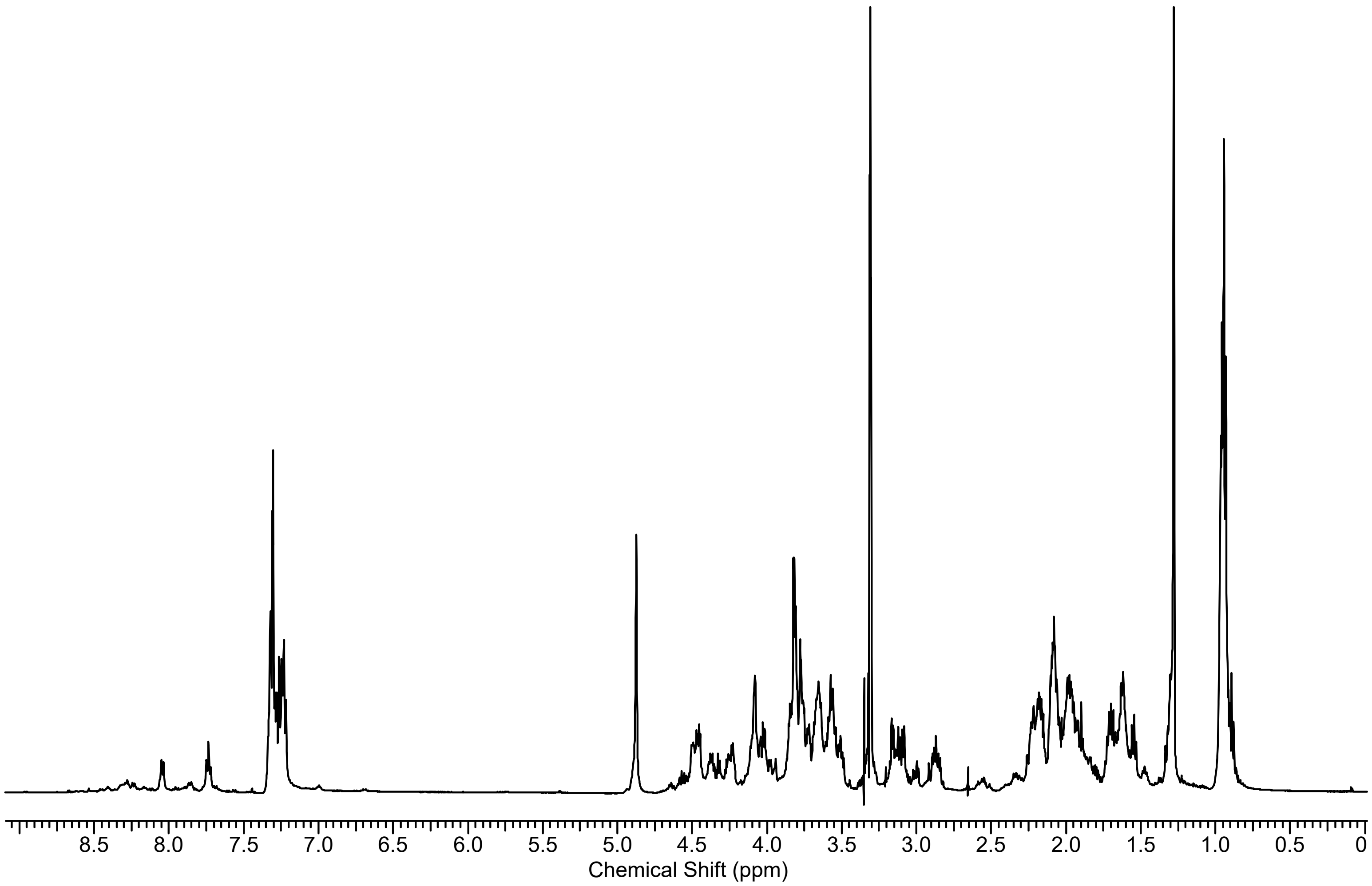

Figure S8. ^{1}H-NMR (500 MHz, CD_3OH) spectrum of cellulamide B (**2**)

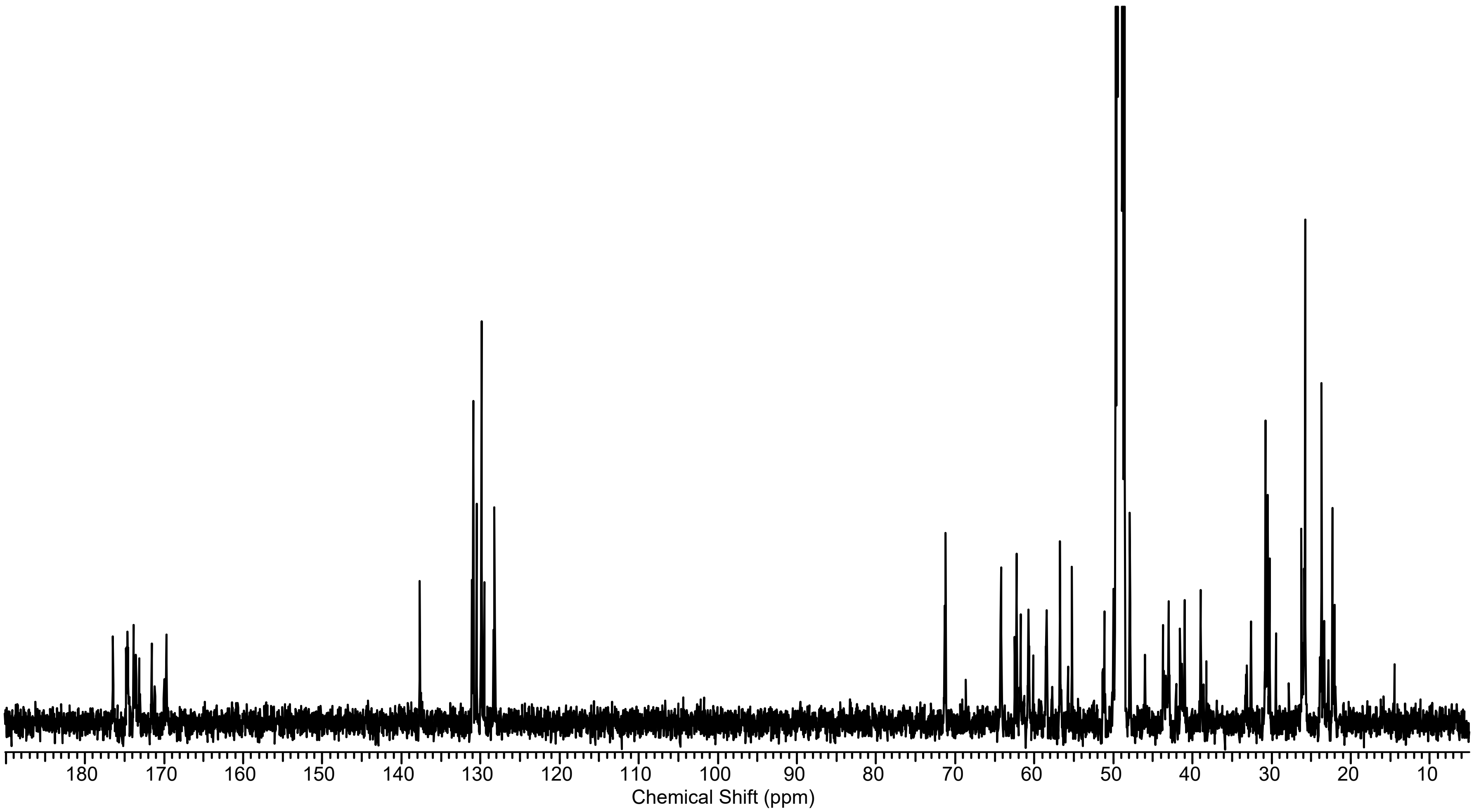

Figure S9. ^{13}C-NMR (125 MHz, CD_3OH) spectrum of cellulamide B (**2**)

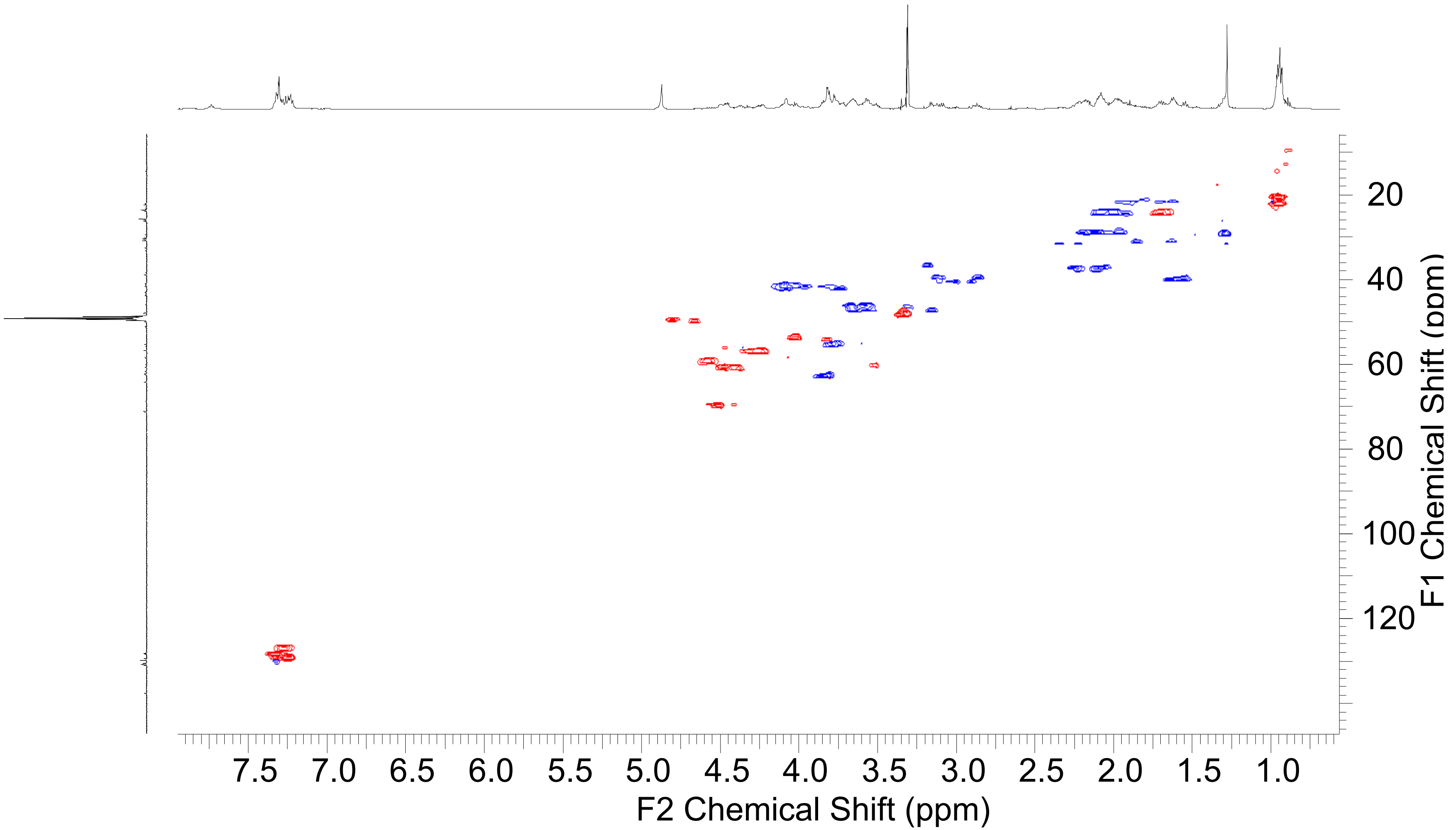

Figure S10. HSQC (CD3OH) spectrum of cellulamide B (**2**).

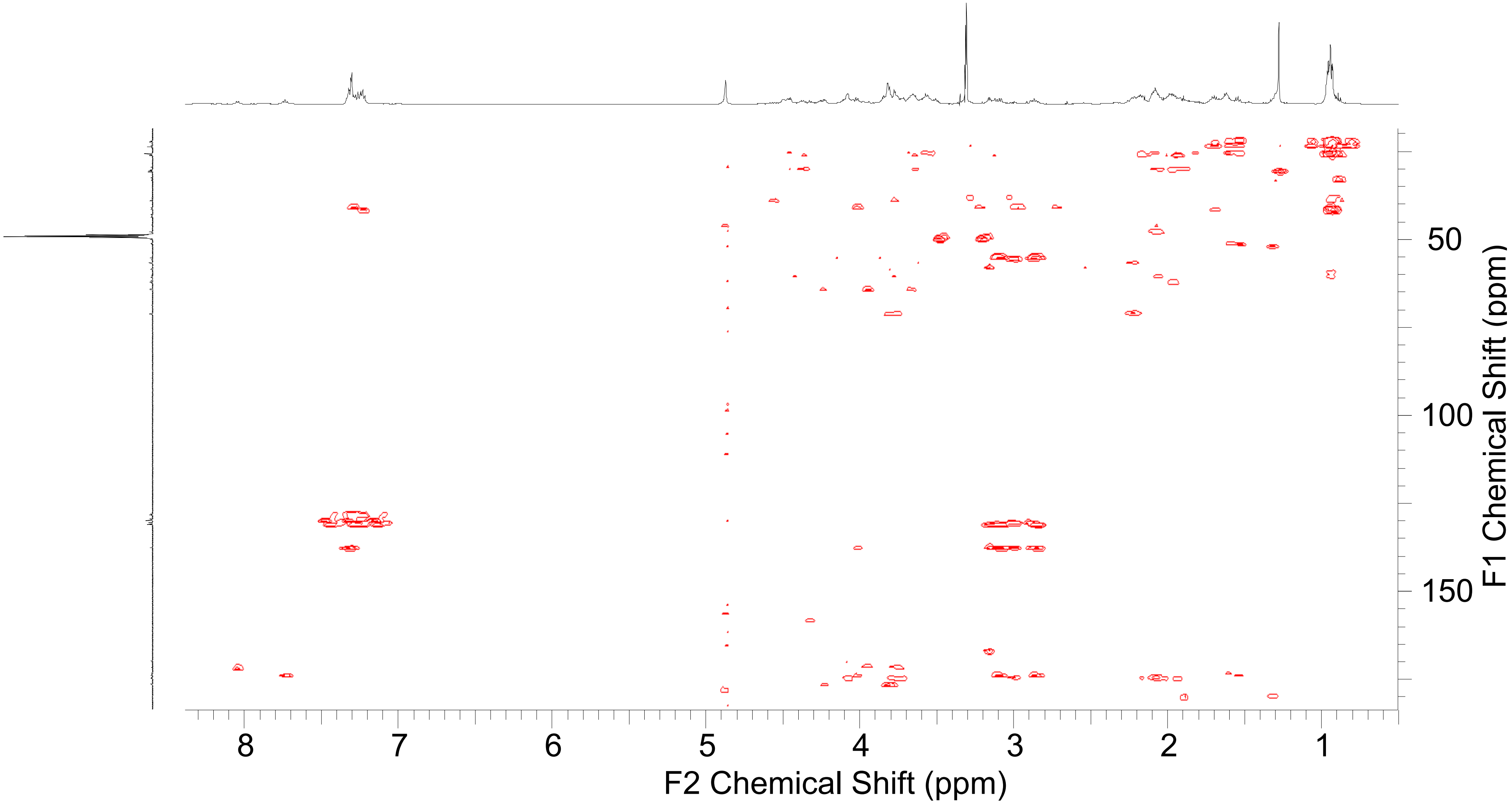

Figure S11. HMBC (CD_3OH) spectrum of cellulamide B (**2**)

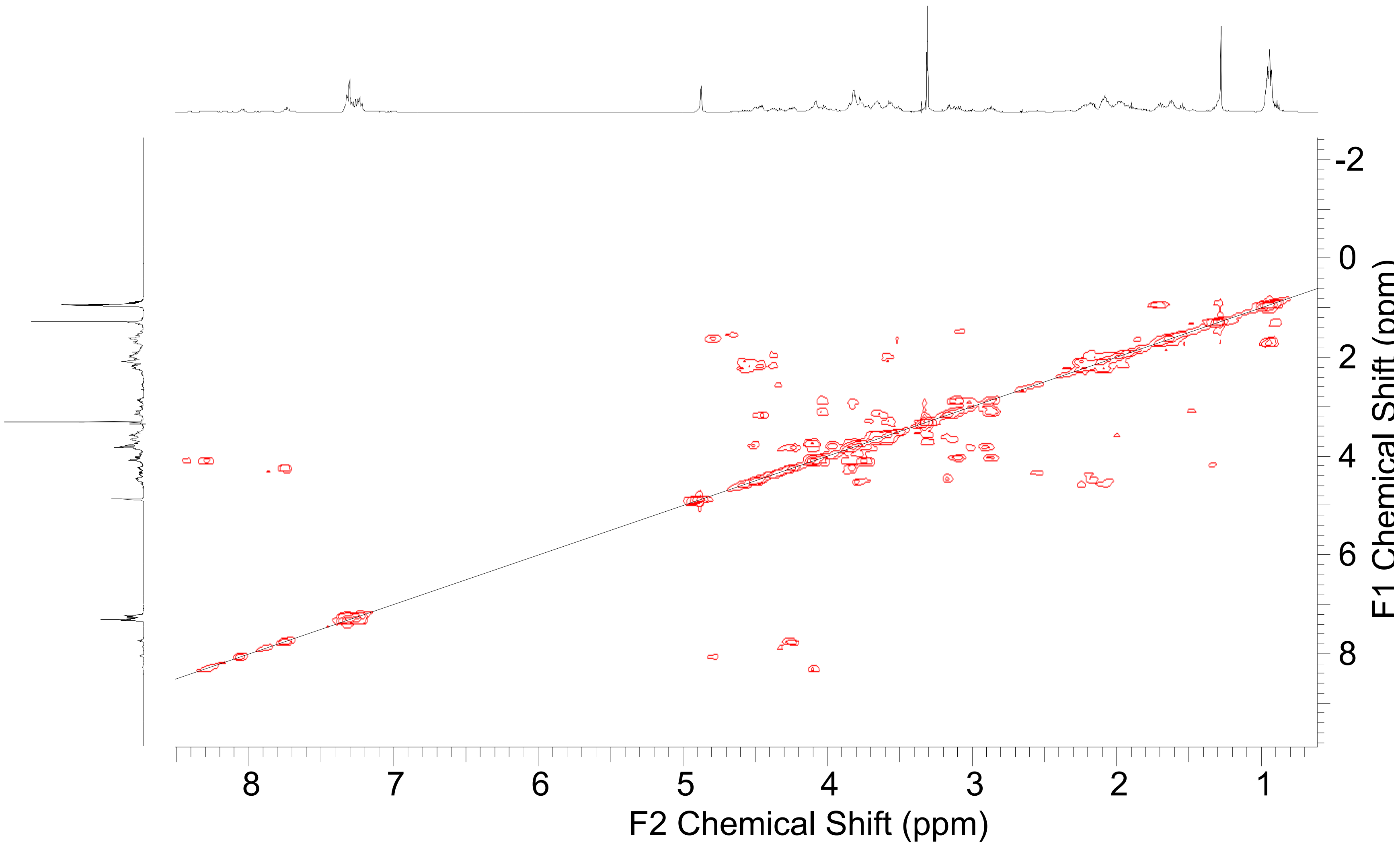

Figure S12. COSY (CD_3OH) spectrum of cellulamide B (**2**)

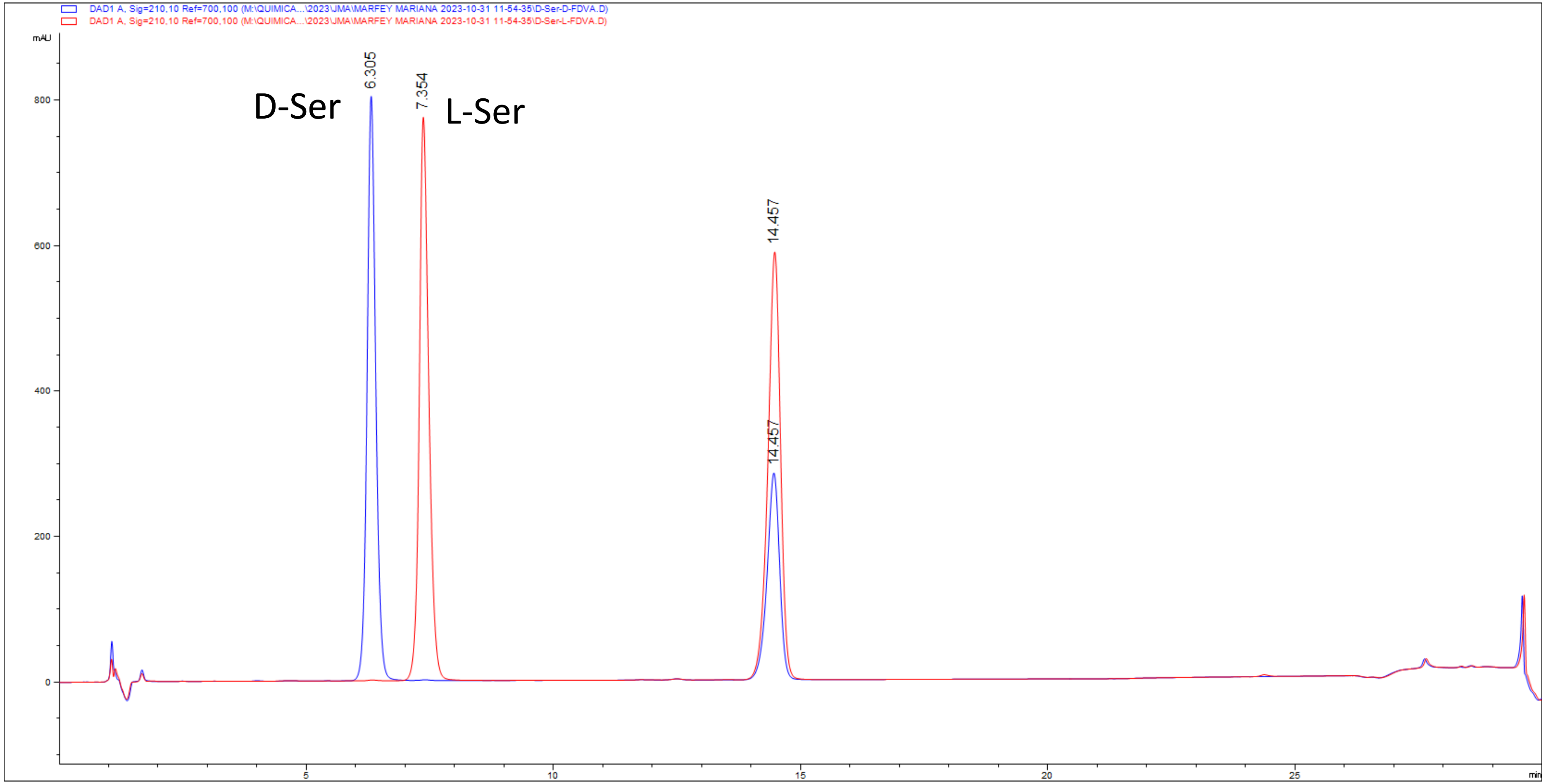

Figure S13. HPLC traces at 210 nm of the D-FDVA derivatives of Ser

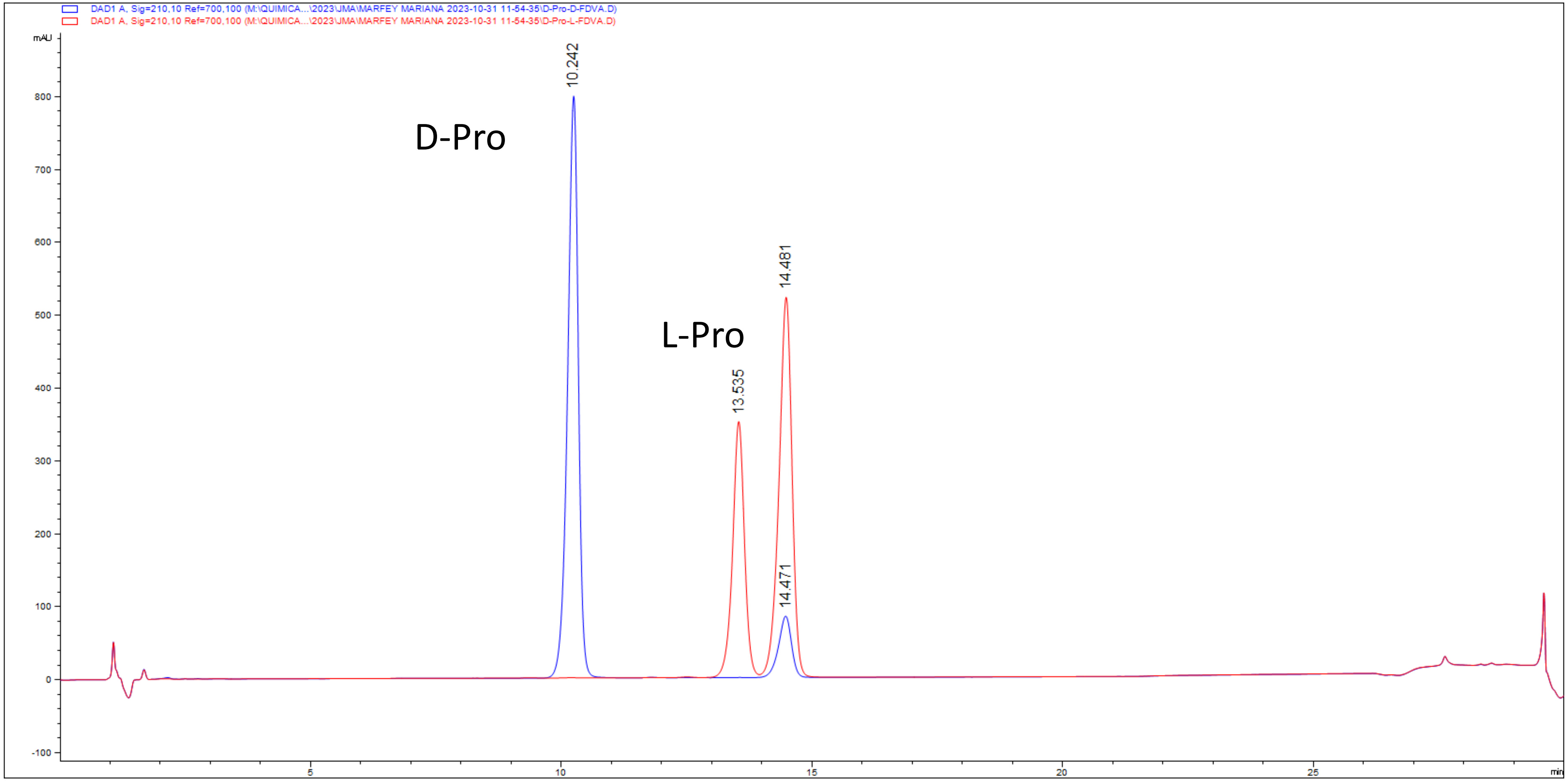

Figure S14. HPLC traces at 210 nm of the D-FDVA derivatives of Pro

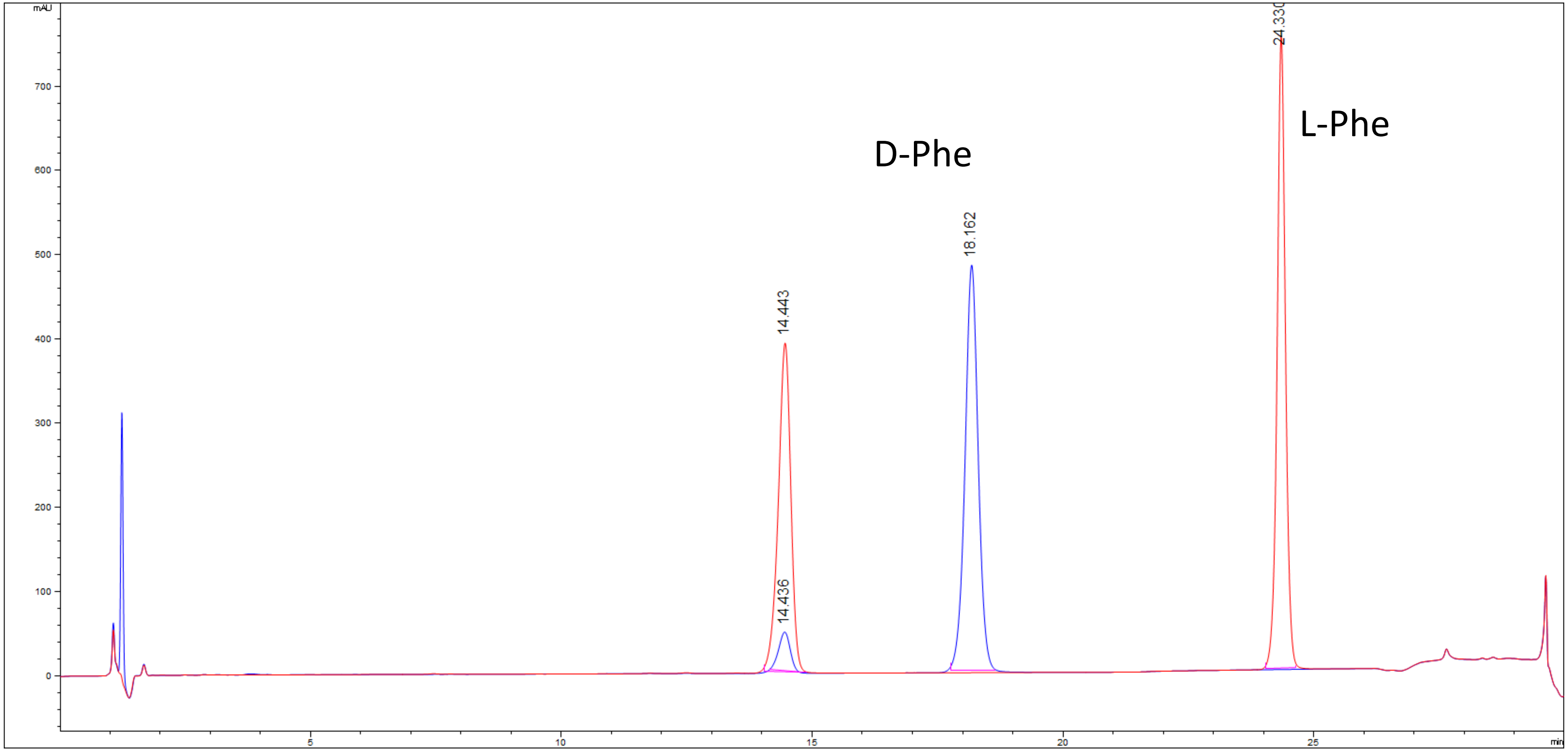

Figure S15. HPLC traces at 210 nm of the D-FDVA derivatives of Phe

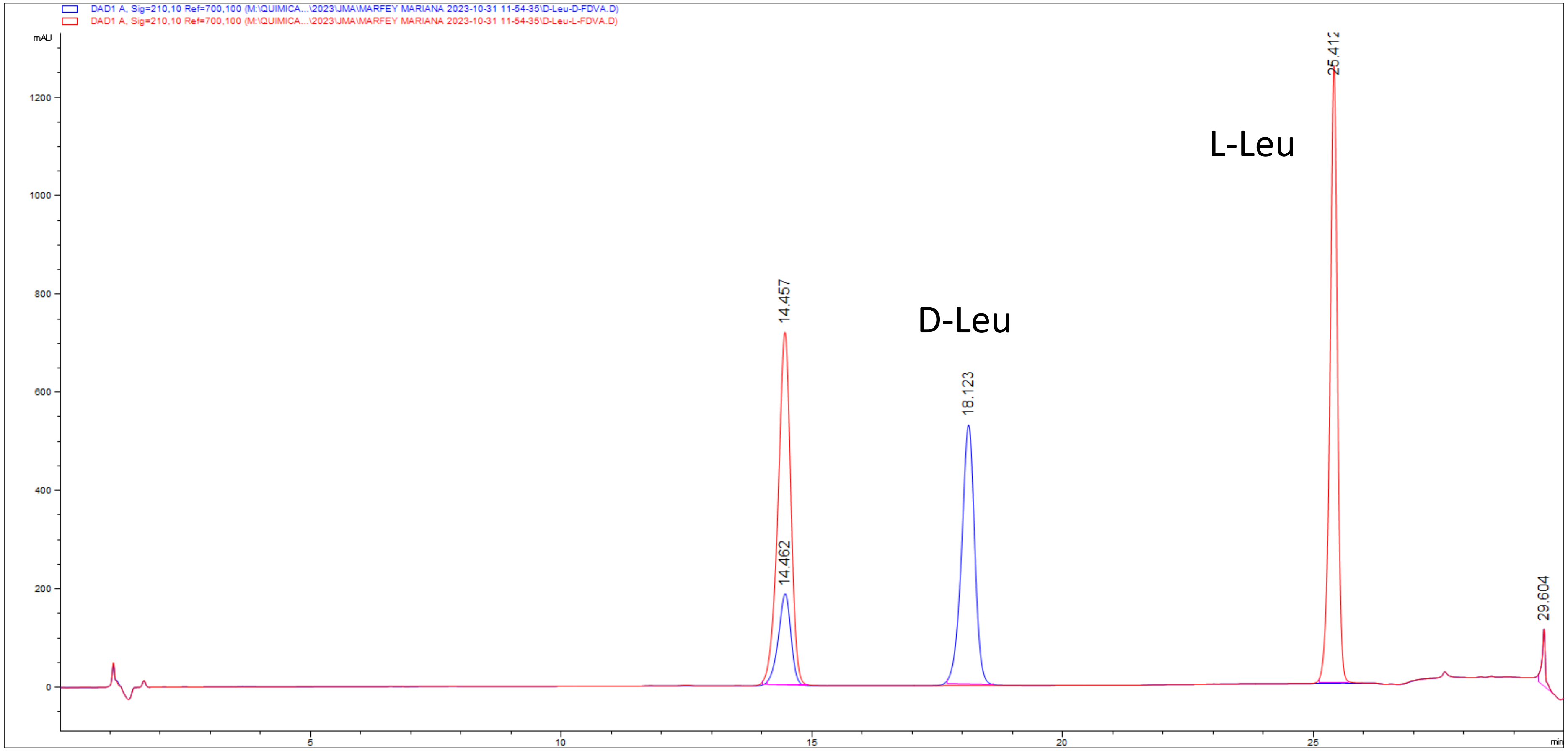

Figure S16. HPLC traces at 210 nm of the D-FDVA derivatives of Leu

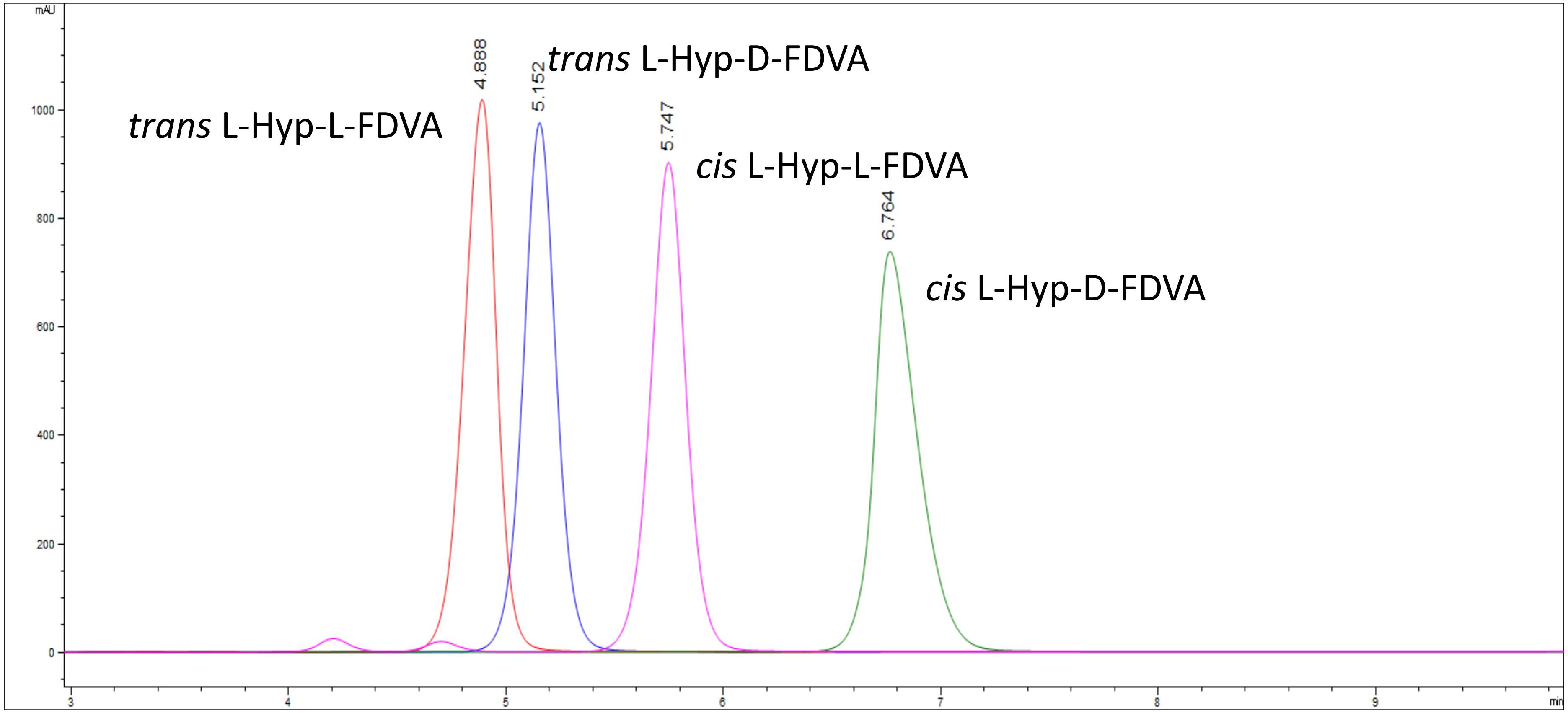

Figure S17. HPLC traces at 210 nm of the D and L-FDVA derivatives of *cis* and *trans* L-Hyp

trans L-Hyp-D-FDVA

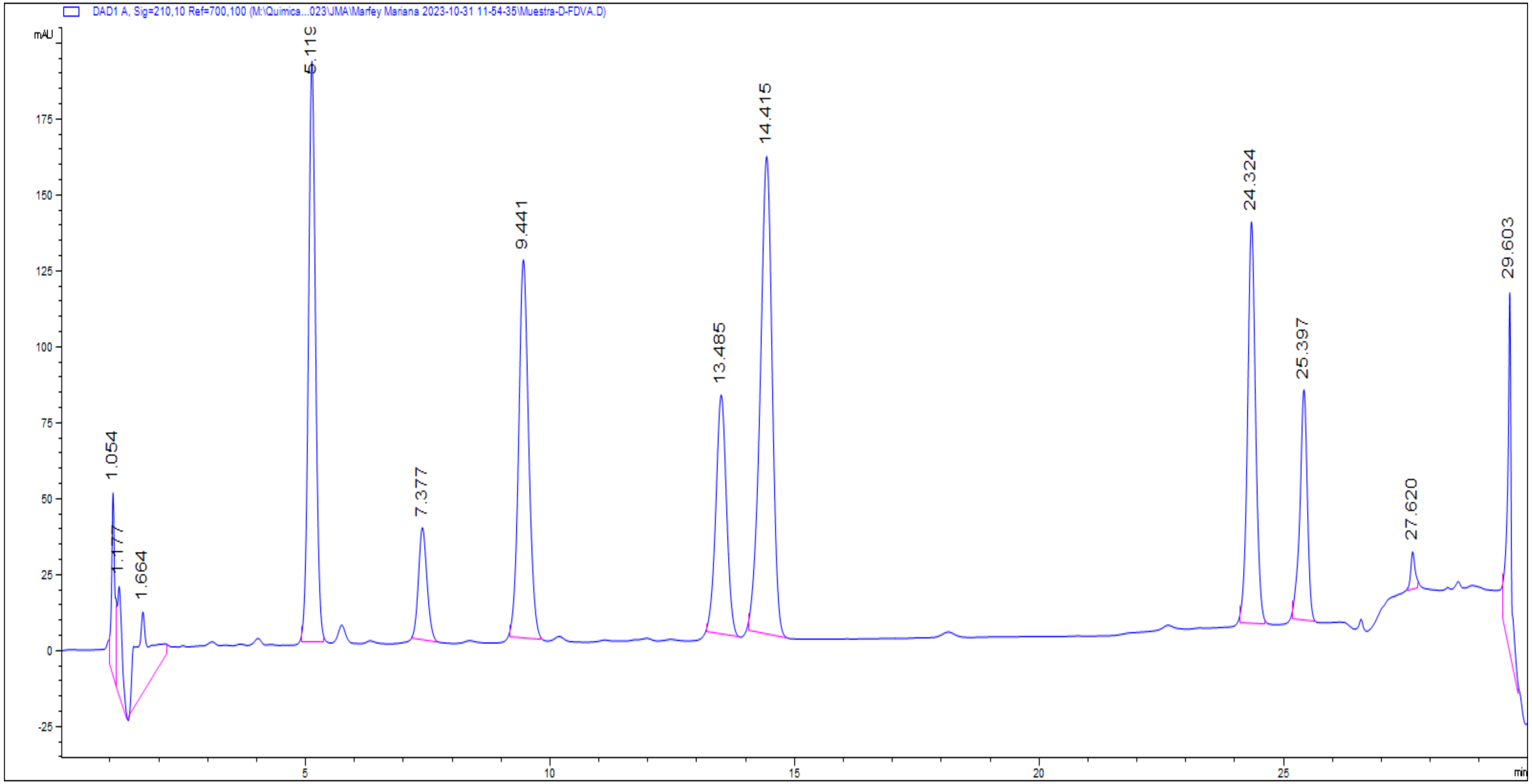

Figure S18. HPLC trace at 210 nm of the D-FDVA derivatives of the hydrolyzate of cellulamide A (**1**)

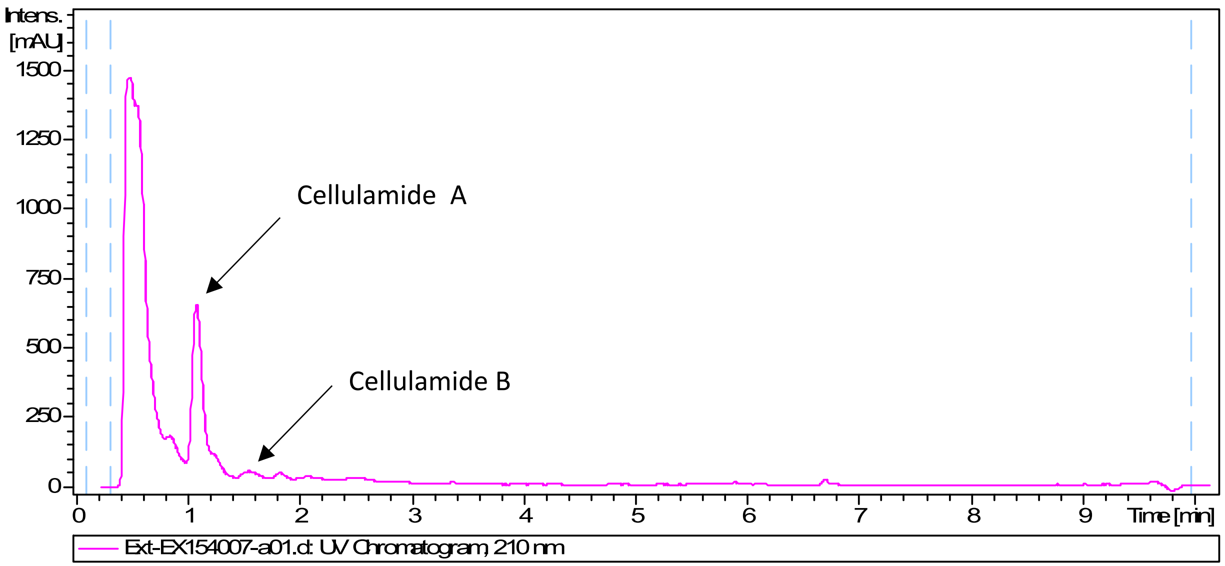

Figure S19. HPLC trace at 210 nm of the D-FDVA derivatives of the hydrolyzate of cellulamide A (**1**)
